# Borate-Based Bioactive Glass Powders for 3D Printing of Biomimetic Resorbable Bone Implants

**DOI:** 10.3390/biomimetics11080564

**Published:** 2026-08-07

**Authors:** Yoann Matagne, Guillaume Marchal, Damien Coibion, Sébastien Blasutig, Fanny Lambert, Frederic Boschini, Rudi Cloots, Nicolas Somers

**Affiliations:** 1 Group for Research in Energy and Environment from Materials (GREEnMat), University of Liège, Quartier Agora–B6a, Allée du Six-Août 13, 4000 Liège, Belgium; yoann.matagne@student.uliege.be (Y.M.); guillaume.marchal@uliege.be (G.M.); damien.coibion@uliege.be (D.C.); frederic.boschini@uliege.be (F.B.); rcloots@uliege.be (R.C.); 2 GeMMe (Georesources, Mineral Engineering & Extractive Metallurgy), University of Liège, 4000 Liège, Belgiumfanny.lambert@uliege.be (F.L.)

**Keywords:** bioactive glass, 3D printing, biomimetic scaffolds, bone regeneration

## Abstract

As the population ages, the demand for customizable, resorbable bone implants in tissue engineering has intensified, outstripping the limitations of traditional autografts and allografts. While silicate-based bioactive glasses dominate bioactive glass research, borate-based bioactive glasses (BBGs) present distinct biomimetic advantages due to their accelerated degradation kinetics and superior ion-release profiles. However, producing highly pure, homogeneous BBG powders tailored for additive manufacturing remains a severe bottleneck. This study reports the development of a highly efficient synthesis protocol and subsequent Digital Light Processing (DLP) 3D printing of BBG scaffolds. An aqueous-based precursor mixture was processed via spray drying and a customized multi-stage thermal pretreatment sequence up to 800 °C to mitigate material loss, minimize oxide evaporation, and completely eliminate carbonates. Subsequent “flash melting” at 1150 °C for 20 min yielded an amorphous, high-purity borate–phosphate glass network (68.1B_2_O_3_-3.8Na_2_O-18.9CaO-4.9MgO-4.3P_2_O_5_, in wt%). Differential scanning calorimetry (DSC) revealed a glass transition temperature ($T_{g}$) of 625 °C, while in situ X-ray diffraction localized the onset of crystal nucleation between 706 °C and 723 °C. Following fine planetary milling to achieve a highly dense particle packing distribution (D_v_50 = 5.4 µm, D_n_50 = 0.6 µm), the optimized BBG powder was successfully loaded into an acrylate-based photosensitive slurry (51.2 wt% solid loading) to manufacture complex 3D biomimetic gyroid scaffolds via DLP. While the structural feasibility of printing high-resolution gyroid porous architectures is validated, post-printing evaluation highlighted a narrow thermal processing window; sintering at 660 °C optimized particle coalescence while minimizing microstructural de-densification caused by closed porosity expansion (which reaches 48.4% at 675 °C). This scalable synthesis-to-printing workflow offers a crucial steppingstone toward next-generation fully resorbable bone tissue scaffolds.

## 1. Introduction

As the population steadily grows and ages, the medical and scientific community faces a growing demand for effective and innovative solutions in tissue engineering and regenerative medicine solutions. Alongside aging, modern lifestyle deteriorates the situation. Poor nutrition, chronic stress, limited physical activity and exposure to harmful substances, such as tobacco, alcohol, industrial cosmetics and environmental pollutants, all contribute to the prevalence of complex diseases including diabetes, cardiovascular disorders, osteoporosis, degenerative diseases and cancer [1,2]. These lead to a significant increase in complex defects, challenging the natural healing process of our body.

Autografts and allografts have long been the traditional gold standards for tissue repair. However, they present respective notable limitations: limited donor tissue availability, invasive harvesting procedures, risks of immune rejection and disease transmission, and difficulties in shaping the grafts to specific needs [3].

In this context, it becomes essential to develop more versatile, accessible and customizable biomaterials. Materials that would not only be biocompatible, resorbable and promote tissue regeneration, but also easily adapted to different tissues and injuries, offering appropriate properties, such as mechanical strength, degradation rate and ion release. To achieve this, researchers increasingly turn to biomimetic design strategies, which seek to replicate the complex architectural, mechanical, and biochemical cues of the native extracellular matrix to guide cellular behavior and tissue neoformation [4]. The field of biomaterials has significantly evolved over the last decades, progressing from inert and passive implants to active and dynamically interacting substitutes that trigger biological responses and stimulate the healing process [2].

Among these biomaterials, bioactive glasses (BGs) have emerged as particularly promising candidates. Discovered in the 1960s, these innovative materials can readily bond to tissues, stimulate biological activity and tissue growth, making them suitable for a wide range of applications from bone repair to wound healing and drug delivery [5,6]. From a biomimetic standpoint, BGs excel because their chemical composition and dissolution products closely mimic the inorganic phase of natural bone, triggering a cascade of physiological ions that actively signal cells to regenerate tissue [5]. Silicate-based BGs have been the most widely studied and applied type of bioactive glasses, but recent research is now looking at alternative compositions. One option that has seen growing interest is borate-based bioactive glasses (BBGs). By replacing the traditional silica network with boron oxide, these glasses offer faster degradation rates, enhanced ion release, and more complete degradation, making them promising solutions for applications where rapid interaction and full resorption are required [7,8,9]. Beyond their composition, the shaping of bioactive glasses into scaffolds is also crucial. In this context, additive manufacturing techniques are emerging as powerful tools for designing elaborate, specific and porous scaffolds, improving the potential of bioactive glasses. Crucially, 3D printing enables the fabrication of scaffolds that mimic the hierarchical porosity, interconnected networks, and anisotropic mechanical structures found in native trabecular and cortical bone, bridging the gap between synthetic constructs and natural anatomy [4,10,11,12,13,14].

Although continuous progress has been made and promising results are being obtained, the field still faces several challenges [12,13,14]. First, the main approaches to synthesize bioactive glasses, the melt-quenching technique and the sol–gel route, present non-negligible drawbacks. The melt-quenching technique requires high temperatures and often results in poor homogeneity, purity and porosity, while the sol–gel route involves costly and harmful reagents while being time-consuming. Sol–gel processes require the elimination of organic and/or nitrate residues through heat treatments to prevent any toxicity of the bioactive glasses. These treatments can potentially lead to unwanted crystallization, which can hinder the BGs bioactivity and integration [12,15,16]. Because of these limitations, commercially available bioactive glasses remain very expensive, varying from 600 €/kg (Nano Research Elements, Singapore) to 10,000 €/kg (Noraker, France) [17]. Moreover, no borate-based bioactive glasses are currently available on the market [17]. These constraints motivate researchers to develop innovative BG synthesis processes, aiming for a more accessible, reproducible, and scalable alternative adapted to additive manufacturing needs.

A second challenge concerns the poor mechanical properties of bioactive glasses which limit their use in load-bearing applications [7,17,18]. Currently, there is no bioactive glass scaffold available commercially because researchers struggle to produce scaffolds that are strong and porous at the same time. Complete densification of the glass framework is necessary for maximum strength and crack resistance. Unfortunately, most BGs, especially the popular 45S5, produce weak scaffolds prone to cracking because they do not sinter to full density. Strategies to increase these properties include combining them with mechanically stronger materials, such as hydroxyapatite (HAP) or some polymers (polylactic acid (PLA) or polycaprolactone (PCL) for instance), through additive manufacturing [19,20]. However, a significant amount of BGs is required to ensure bioactivity in each scaffold and for testing. Furthermore, physicochemical properties of commercial powders are not always optimized for 3D printing applications (i.e., particle size and morphology). This further supports the need for further development of production methods.

The primary objective of this study is to develop a reliable, cost-effective, and reproducible laboratory-scale production workflow for high-purity borate-based bioactive glass powders customized specifically for slurry-based vat photopolymerization. This study seeks to address critical manufacturing challenges, namely debinding and sintering, required to yield functional, fully resorbable bone implants for further regenerative medicine evaluation. To overcome the low homogeneity and significant oxide evaporation characteristic of conventional melt-quenching methods [8], which typically require prolonged high-temperature processing that shifts the intended chemical composition of volatile borate networks [21], this work bridges a critical synthesis-to-manufacturing gap. An alternative, highly reproducible workflow combining automated aqueous spray drying with a rapid “flash-melting” technique has been engineered. While the prior literature has used solution chemistry primarily for low-temperature sol–gel glasses [21], the approach of this paper exploits spray drying to achieve sub-micron, homogeneous and intimate mixing of chemical precursors. This structural intimacy drives down the required thermal holding period at 1150 °C to just 20 min, effectively minimizing the volatilization kinetics of B_2_O_3_ and P_2_O_5_ fractions while completely eliminating toxic nitrate/carbonate intermediates prior to slurry processing.

Furthermore, this research intends to exploit these synthesized powders to fabricate complex scaffolds via Digital Light Processing (DLP). To guide tissue neoformation, a triply periodic minimal surface (TPMS) gyroid architecture was chosen to fulfill a three-fold biomimetic strategy:

- Structural biomimicry: The continuous gyroid architecture replicates the high interconnectivity, zero-mean curvature, and open macroporosity (macropores around 330 μm) characteristic of native trabecular bone [22,23].
- Compositional Biomimicry: The constituent oxides within the glass network (CaO, MgO, P_2_O_5_, and the minor SiO_2_ phase) closely resemble the essential chemical building blocks of the inorganic mineral phase of natural bone [12,13].
- Biological Biomimicry: Upon exposure to biological fluids, the rapidly dissolving glass releases a physiological ionic cascade (Ca^2+^, PO_4_^3−^, BO_3_^3−^, Mg^2+^, Si^4+^) that stimulates rapid surface mineralization, precipitating a biomimetic bone-like apatite layer [12,16].

By thoroughly investigating the thermal behavior, structural integrity, in vitro apatite-forming capability, and rheological suitability of the glass-loaded resin, this study seeks to address the critical manufacturing challenges, namely debinding and sintering, required to yield functional, fully resorbable bone implants for regenerative medicine.

## 2. Materials and Methods

### 2.1. Synthesis of Borate-Based Bioactive Glass

The overall synthesis of the borate-based bioactive glass includes precursor mixing and spray drying followed by a thermal pretreatment, a flash melt quenching, then a milling step.

The synthesis procedure relies on two references: Aqdim et al., 2022 [8] and Youssif et al., 2019 [21]. The first one is used as the main reference (reagents, mixing, pretreatment and melt quenching) while the second provides an alternative aqueous mixing method. The composition of the BBG is adapted from [8]. A low sodium oxide content is chosen to increase the onset crystallization temperature, a critical parameter during the debinding and sintering process of the printed scaffolds. The targeted final composition of the glass is, in wt%, 68.1B_2_O_3_-3.8Na_2_O-18.9CaO-4.9MgO-4.3P_2_O_5_.

#### 2.1.1. Precursor Mixing

Chemicals used in this work are listed in Table 1.

The reagents are added one by one to Milli-Q^®^ water at 50 °C at 30 min intervals, then vigorously stirred for an additional 1 h. The order of addition is the following: B(OH)_3_, Na_2_CO_3_, CaCO_3_, MgCO_3_, and (NH_4_)_2_HPO_4_. The final precursor-to-water ratio is set to approximately 1:1 to obtain a fluid suspension suitable for spray drying. To optimize the homogeneity and break larger particles, the suspensions are ground using an Ultra-Turrax (IKA^®^ T18 Digital, IKA-Werke GmbH & CO. KG, Germany) for 30 min at 15 krpm. The final suspension is then sieved with a 500 µm sieve before spray drying to avoid any clogging during injection.

#### 2.1.2. Spray Drying

The spray dryer used in this work is the MOBILE MINOR^®^ R&D Spray Dryer (GEA Spray Dryers, Düsseldorf, Germany). The solid loading of the suspension is set to 50 wt% and the parameters used are displayed in Table 2.

#### 2.1.3. Thermal Pretreatment

Before melting, the spray-dried mixture undergoes thermal pretreatment to decompose the reagents and remove residual moisture. This step prevents gas-releasing reactions from occurring during melting, which can lead to foaming and material loss and ensures complete homogenization of the melt.

**Figure 1:**
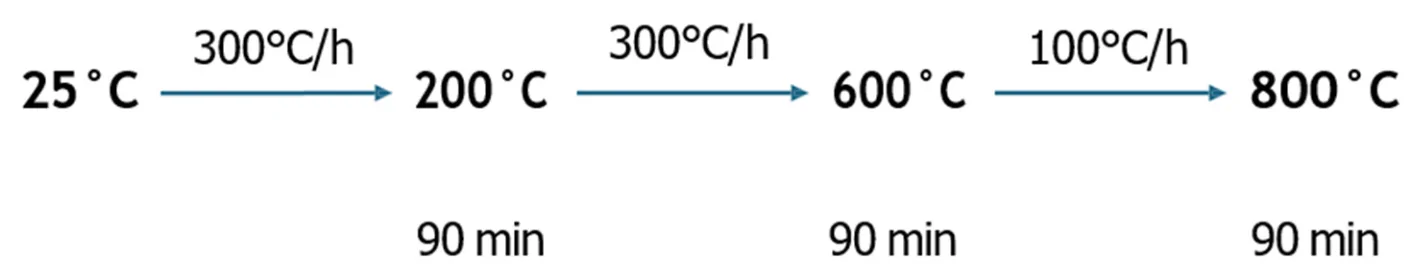
Heating profile of the precursor pretreatment.

The thermal treatment described in Aqdmin et al., 2022 [8] is optimized based on the reagents decomposition temperatures found in the literature [24,25] and on the TGA-DSC results obtained for the precursor mixture (see Section 3.1 Synthesis of borate-based bioactive glass—mix of precursors and discussion). The pretreatment of the precursor is carried out in a porcelain crucible under an air atmosphere. The precursor mixture is heated up to 800 °C according to the temperature profile shown in Figure 1. During this treatment, the dwell at 200 °C ensures the removal of residual moisture and decomposition of B(OH)_3_ and (NH_4_)_2_HPO_4_ into their corresponding oxides. The plateau at 600 °C allows partial decomposition of sodium and magnesium carbonates, reducing CO_2_ release when B_2_O_3_ and P_2_O_5_ are molten, thus reducing the foaming effect. The final dwell time at 800 °C ensures complete decomposition of all remaining carbonates.

The heating ramps and dwelling durations are selected and experimentally validated via the physical milestones identified in our TGA-DSC analysis (Section 3.1) to limit gas emissions, suppress raw precursor foaming, and minimize $P_{2}O_{5}$ evaporation:

- From 25 °C to 200 °C at 300 °C/h with a 90 min dwell: This window directly addresses the initial volatilization region below 250 °C. It isolates the evaporation of physical water alongside the early thermal breakdown of B(OH)_3_ and (NH_4_)_2_HPO_4_. Extending this dwell to 90 min ensures that the high volume of generated H_2_O vapor and NH_3_/NO_x_ streams gas off smoothly without generating fluid macro-bubbles that trigger raw material ejection from the porcelain crucible.
- From 200 °C to 600 °C at 300 °C/h with a 90 min dwell: This step limits P_2_O_5_ evaporation—which typically exhibits high vapor pressures between 300 °C and 360 °C—by favoring its structural capture and chemical incorporation into the softening B_2_O_3_ glass former matrix. Simultaneously, this intermediate dwell safely drives the partial decomposition of the lower-temperature magnesium and sodium carbonates.
- From 600 °C to 800 °C at a slower rate of 100 °C/h with a 90 min dwell: Our TGA curve (Section 3.1)) isolates this window as the primary zone for CaCO_3_ calcination. Slowing the heating rate down to 100 °C/h controls the kinetics of this high-volume CO_2_ release. Holding at 800 °C ensures complete calcination immediately prior to the system undergoing the major exothermic network rearrangement observed at 818 °C, where calcium/magnesium borates and mixed phosphate networks crystallize. By completing all gas-releasing structural reactions before these rigid crystal phases lock up the matrix, the resulting calcined mass can be melted rapidly at 1150 °C without experiencing any crucible foaming, bubbling defects, or compositional drift.

The mixture is then allowed to cool naturally to room temperature and manually ground in a porcelain mortar.

#### 2.1.4. Melt Quenching

Quartz crucibles coated with a boron nitride (BN) aerosol (SL-E 125, HeBoCoat^®^ Henze, BNP AG, Lauben, Germany) prior to melting to limit degradation and potential contamination are used for the melt-quenching synthesis [24,25]. The pretreated powder is first placed in an oven preheated at 600 °C in the BN-coated quartz crucible for 30 min to limit thermal shocks. Then, the powder is manually stirred to break any eventual agglomerates and quickly transferred into a furnace preheated to 1150 °C. The crucible is then held for 20 min at 1150 °C to ensure a homogeneous melting of the powder. This “flash-melting” approach reduces the time spent at high temperature and therefore limits evaporation of B_2_O_3_ and P_2_O_5_ fractions, ensuring the desired oxide composition. The molten glass is then rapidly quenched by pouring it onto a steel plate at room temperature and pressing it by a second one covered with 1 kg of ice. This rapid cooling prevents crystallization and ensures a resulting amorphous glass.

#### 2.1.5. Milling

A planetary ball mill PM 400 from Retsch (Haan, Germany) is used to grind the obtained bulk glass after melt quenching into fine powder suitable for 3D printing. The powder is milled at 400 rpm (2 × 15 min), with a 15 min cooling interval in an ice bath between cycles, in zirconia jars using zirconia balls of 5, 10, and 20 mm diameter (approximately 1:1:1 mass ratio) in Milli-Q water. The glass:water:beads ratio is fixed at 1:1:7. The powder is recovered through a 500 µm sieve and by Buchner filtration, then dried at 80 °C overnight and finally ground by hand in a porcelain mortar.

The experiments have been repeated 3 times and statistical analyses were performed (arithmetic mean and standard deviation) for numerical values.

### 2.2. Characterizations of the Borate-Based Bioactive Glass Powder

The composition of the material is investigated by X-ray diffraction (XRD), X-ray fluorescence (XRF) and Fourier-Transform Infrared Spectroscopy (FTIR) techniques. The morphological properties and particle size are characterized by laser diffraction granulometry and scanning electron microscopy (SEM). Thermogravimetric Analysis (TGA) and differential scanning calorimetry (DSC) are used to assess the thermal behavior of the material.

#### 2.2.1. X-Ray Diffraction

X-ray diffraction measurements are performed in Bragg–Brentano geometry using a Bruker (Billerica, MA, USA) D8 Twin–Twin diffractometer with Cu Kα radiation and Lynxeye XET 1D detector. Powders are analyzed over the 2θ range from 10° to 70°, with 0.2 s/channel step time and 15 rpm rotation speed. The whole powder pattern fitting procedure is performed using TOPAS Academic Version 8 and Profex 5.7 [26] softwares. The fundamental parameters approach is used to describe the instrumental contribution [27]. The crystalline phases are identified using Joint Committee on Powder Diffraction International Center for Diffraction Data (JCPDS-ICDD) files. In addition, the crystallization process is monitored through in situ XRD measurements at a 10 °C/min heating ramp until 800 °C in the 20–35° 2θ region.

#### 2.2.2. X-Ray Fluorescence

XRF analyses are performed by the GeMMe laboratory using a Bruker S8 TIGER spectrometer. The measurements are carried out on pressed powder pellets, prepared by compacting the dried powder into mechanically stable disks (12 mm of diameter), and are used to determine the elemental composition of the final glasses.

#### 2.2.3. Fourier-Transform Infrared Spectroscopy

The analyses are performed using the Attenuated Total Reflectance (ATR) mode, which allows direct measurement of powders without specific preparation. FTIR measurements are carried out on a Nicolet iS5 (Thermo Fisher Scientific, Waltham, MA, USA) spectrometer equipped with an iD7 ATR module, over the range 500–4000 cm^−1^, and each spectrum corresponds to the average of 32 scans.

#### 2.2.4. Thermogravimetric Analysis (TGA) and Differential Scanning Calorimetry (DSC)

TGA and DSC analyses are done simultaneously using the LabSys evo (Setaram, Caluire-et-Cuire, France) with a heating ramp of 10 °C/min in air. Both borate-based bioactive glass powder and green 3D part are characterized.

#### 2.2.5. Laser Diffraction Granulometry

Particle size distributions are obtained using a Malvern (Worcestershire, UK) Mastersizer 3000 equipped with a Hydro EV liquid cell. Measurements are performed in deionized water (refractive index = 1.33), using silica as the optical model for the particles (refractive index = 1.457). The suspension is stirred at 2500 rpm, and the powder concentration is adjusted to reach a target obscuration of 2–12%. For each sample, three consecutive measurements are recorded and averaged to obtain the final particle size distribution.

#### 2.2.6. Scanning Electron Microscopy

A field emission gun microscope TESCAN (Brno, Czech Republic) CLARA equipped with an energy dispersive X-ray spectroscopy (EDX) detector, processed under a 15 kV accelerating voltage and high vacuum, is used for the morphological characterization of powders. The powders are deposited on carbon tapes. The sputtering deposition is done with carbon fibers under vacuum (SPI-MODULE Carbon Coater, SPI Supplies, West Chester, PA, USA). 3D parts are also characterized by SEM to assess the presence of defects.

#### 2.2.7. Simulated Body Fluid (SBF)

Bioactivity of BGs is related to the bonding of the material to living organs through a layer of hydroxyapatite. This HAP-forming ability of the BGs can be assessed with simulated body fluid (SBF) solutions. In these tests, BGs samples are soaked in a solution with a similar ionic composition to human blood plasma for several amounts of time. SBF solutions are prepared following the operating procedure from ISO 23317:2014 [28].

The synthetized and non-sintered BBG powder is pressed into 16 mm diameter pellets by applying 0.75 g of powder under a 2-ton load for 1 min 30 s using a hydraulic press. The pellets obtained are then broken and sanded to produce fragments of the desired mass. These fragments are then immersed with a ratio of 2 mg/mL of SBF in plastic vials at 37 °C under controlled agitation in an incubator. To maintain a constant ionic composition, the SBF solution is refreshed every 2 days: the samples are rinsed with a volume of Milli-Q^®^ water corresponding to half the SBF volume in the vial, before replacing the SBF solution. Upon collection, the samples are rinsed with a volume of Milli-Q^®^ water equal to the full SBF volume in the vial. Samples are collected after 1, 3, and 7 days and analyzed by SEM to examine changes in the surface morphology and XRD to investigate the formation of HAP. The SBF test is performed 3 times to verify bioactivity observations.

### 2.3. Vat Photopolymerization of Borate-Based Bioactive Glass

Once the bioactive glass is obtained and grounded to the desired grain size, it can be incorporated into a photo-curable matrix to prepare a DLP (Digital Light Processing)-compatible slurry. The formulation is taken from an in-house recipe and includes two acrylate-based photosensitive resins (Water-Wash resin 2.0 and High-Speed resin 2.0, Anycubic, Shenzhen, China) a dispersing agent (Rhodafac^®^ RS-610 E, Syensqo, Belgium) to stabilize the glass particles, and a plasticizer (PEG 300, Sigma-Aldrich) to improve the flexibility of the printed parts. The components of the slurry and their final proportions (in wt%) are listed in Table 3. The preparation of the slurry involves several steps to ensure homogeneous dispersion of the particles and appropriate rheological behavior. To obtain a homogeneous slurry, two pieces of equipment are used: a SpeedMixer SMART DAC 600 (Hauschild SpeedMixer Inc., Hamm, Germany) and a three-roll mill (EXAKT 50i, EXAKT Advanced Tech. GmbH, Norderstedt, Germany).

#### 2.3.1. Rheology

Two rheological tests are carried out to establish the dynamic and static behavior of the slurry under processing conditions. First, a viscosity test is performed to determine if the slurry exhibits a shear-thinning behavior and drops below the critical processing threshold (<5000 mPa∙s) when subjected to the high-shear dynamic forces applied by the printer’s recoating blade operating at a wiping speed of 100 mm/s (corresponding to a shear rate of approximately 100 s^−1^). The second test is a viscoelasticity test conducted in oscillatory mode to isolate the linear viscoelastic region (LVER), the rest storage modulus (G′), and the structural yield stress ($\tau_{y}$). These static parameters serve as a direct indicator of suspension stability, verifying if the internal network possesses sufficient structural strength to eliminate particle sedimentation during the rest periods of the light-exposure sequence [29,30].

Both tests are performed using a parallel-plate (PP) configuration (Anton Paar MCR 102, Graz, Austria). The viscosity test is carried out in rotational mode by applying a continuous shear rate ramp to the slurry, while the viscoelastic test is performed in oscillatory mode by applying a controlled sinusoidal shear.

#### 2.3.2. Digital Light Processing

The prepared BBG slurry is then processed by DLP using a Form 4 printer (Formlabs, Somerville, MA, USA). The printing parameters used are displayed in Table 4.

**Figure 2:**
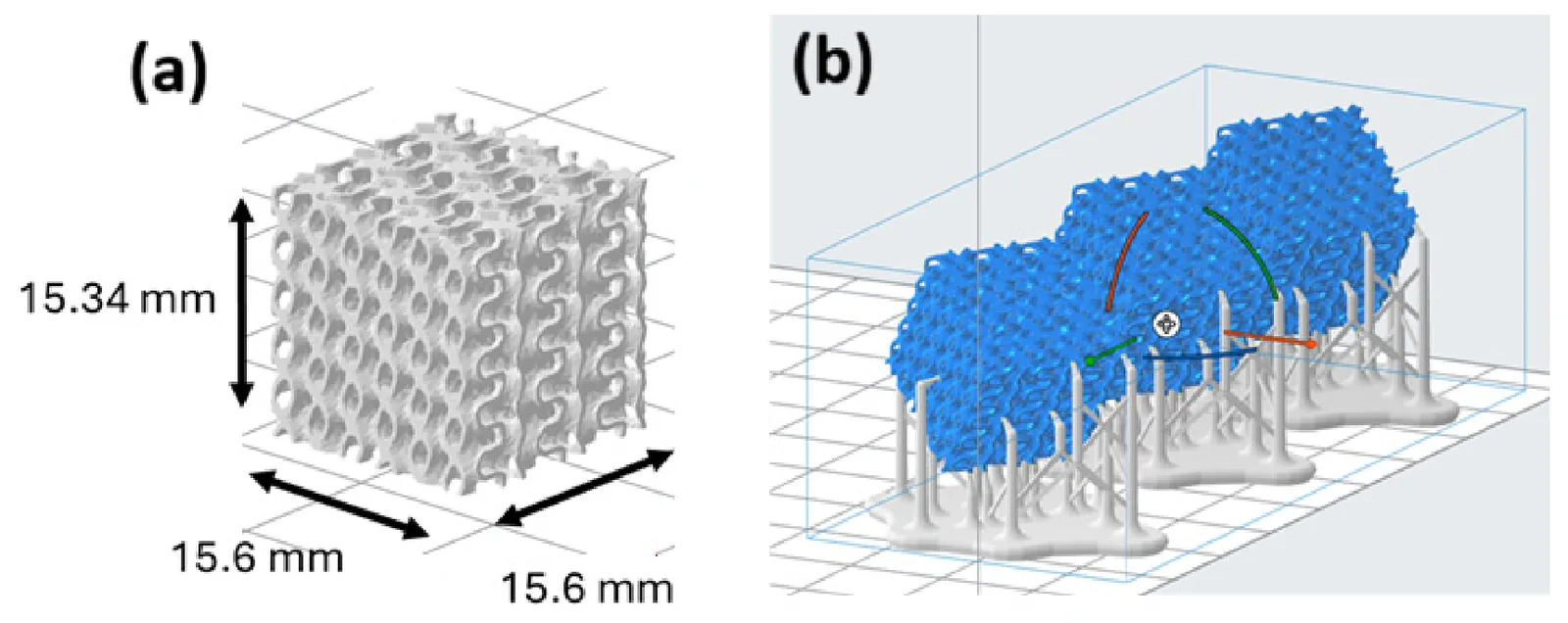
(**a**) Digital model of a gyroid cube used for DLP printing (Cerhum SA [31]); (**b**) file preparation of gyroid cubes with supports (Preform, Formlabs).

The printed scaffolds consist of rectangular parallelepiped (15.60 × 15.60 × 15.34 mm) with a structure based on a gyroid architecture (Figure 2a). This geometry offers a highly ordered and interconnected pore network with large specific surface area, supporting cell colonization [22,23].

The scaffolds are printed diagonally to reduce the contact area with the build platform, helping with detachment after printing. Custom pillar supports are added to improve adhesion during printing and to facilitate scaffolds recovery after printing (Figure 2b). Following the printing step, the green bodies are rinsed with isopropanol to remove uncured slurry and then let dry at room temperature overnight before removing the supports.

#### 2.3.3. Debinding and Sintering

**Figure 3:**
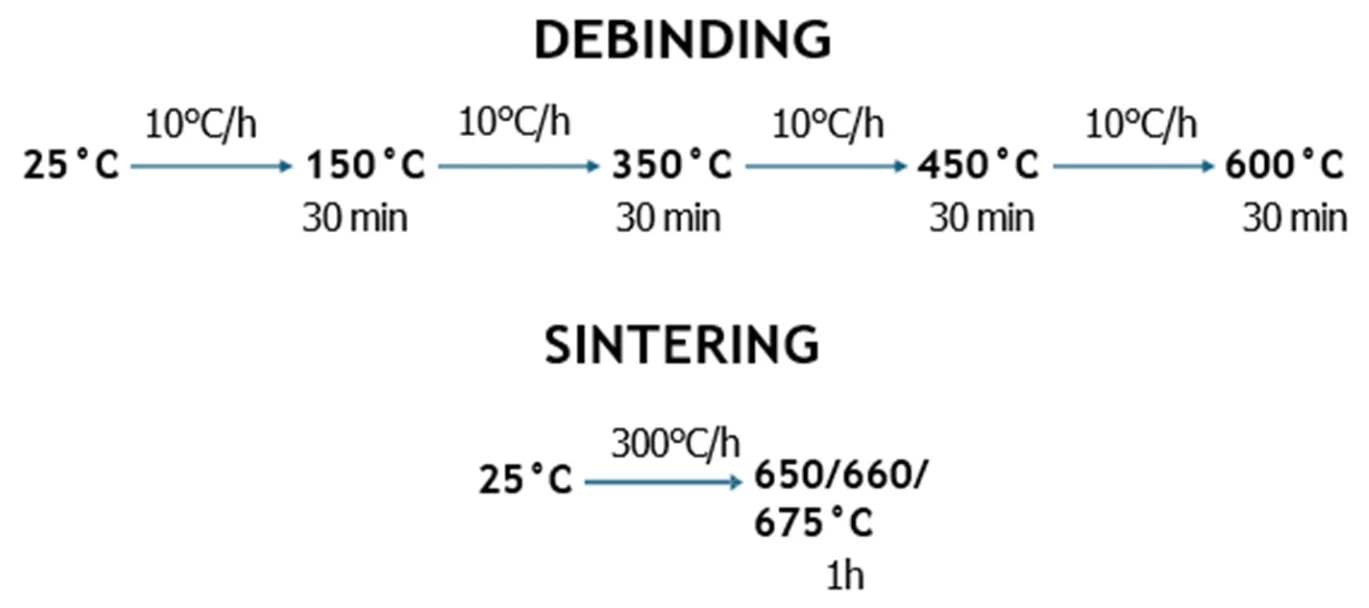
Debinding and sintering thermal cycle.

After printing, the scaffolds undergo a debinding and sintering process (Figure 3), based on TGA-DSC analyses of the obtained BBG powder and on the green bodies, described in Section 3.1. After each cycle, the scaffolds are allowed to cool naturally to room temperature.

### 2.4. Characterizations of 3D Printed Borate-Based Bioactive Glass Parts

#### Density

Relative densities (RDs) as well as open, close and total porosity values of sintered 3D parts are determined by the Archimedes’ method in deionized water at room temperature. The weighing process is conducted on an analytical balance with a resolution of 0.1 mg. A series of 3 samples per composition and temperature are analyzed to obtain average relative density values (theoretical density of 2.5 g/cm^3^) as well as porosity values [31].

## 3. Results and Discussion

### 3.1. Synthesis of Borate-Based Bioactive Glass

#### 3.1.1. Mix of Precursors

**Figure 4:**
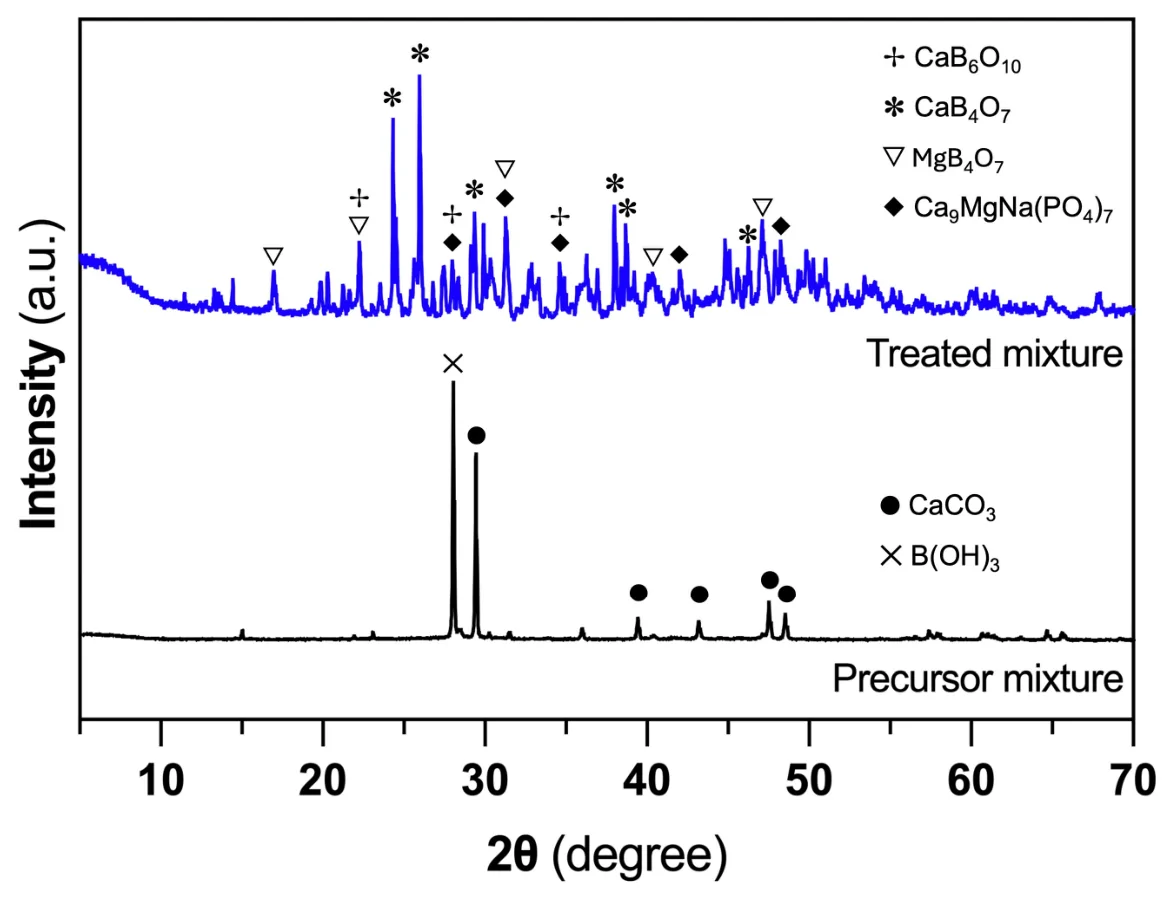
XRD patterns of the spray-dried mixture of precursors before (black) and after thermal pretreatment (blue). CaB_6_O_10_ (PDF 04-016-5636), CaB_4_O_7_ (PDF 00-031-0253), MgB_4_O_7_ (PDF 00-031-0787), Ca_9_MgNa(PO_4_)_7_ (PDF 00-045-0136).

XRD pattern of the spray-dried mixture of precursors is shown in Figure 4 which is mainly characterized by diffraction peaks corresponding to CaCO_3_ and B(OH)_3_. Figure 4 shows the XRD analysis performed on the spray-dried mixture following the thermal pretreatment (800 °C), leading to a complex mix of phases including CaB_6_O_10_, CaB_4_O_7_, MgB_4_O_7_ and Ca_9_MgNa(PO_4_)_7_. These different crystalline phases are the result of the dehydration, decarbonation and decomposition of the spray-dried mix of precursors.

**Figure 5:**
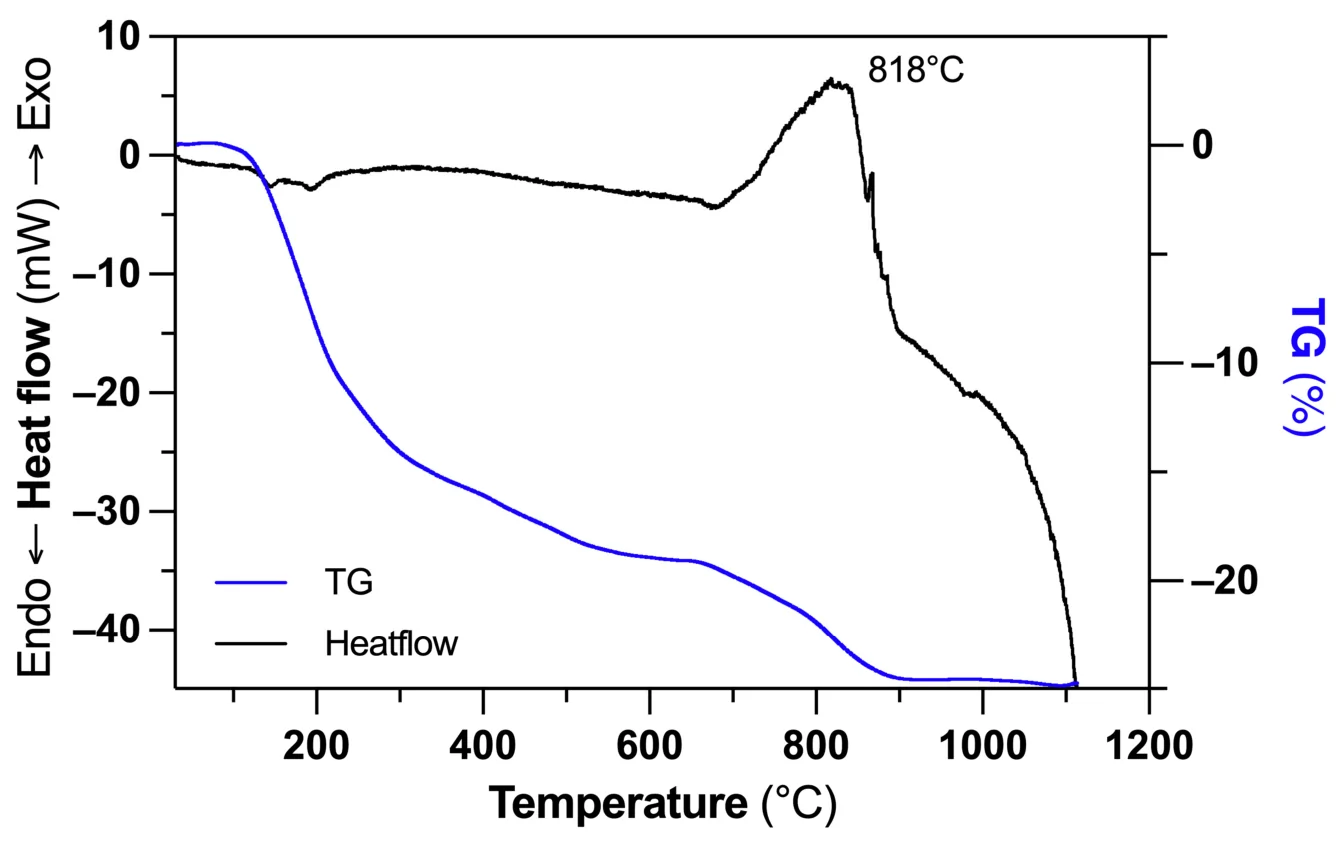
TGA-DSC analysis of the precursor mixture prepared via spray drying.

For a better understanding of the thermal behavior, the TGA-DSC analysis of the precursor mixture prepared via spray drying is shown in Figure 5. The first major mass loss occurs below 400 °C and can be attributed to the removal of adsorbed water, the dehydration and early decomposition of B(OH)_3_ and (NH_4_)_2_HPO_4_ [24]. In this temperature range, assigning the endothermic events is challenging due to overlapping transitions. The first endothermic event at 127 °C is likely associated with the loss of adsorbed water, the second, at 166 °C, with both B(OH)_3_ and (NH_4_)_2_HPO_4_ decomposition [24], and the third, at 208 °C, with the dehydration of (MgCO_3_)_4_Mg(OH)_2_ · 5H_2_O. The diffuse mass-loss step between 370 and 650 °C can be associated with the progressive decomposition of (MgCO_3_)_4_Mg(OH)_2_ · 5H_2_O and decarbonation of Na_2_CO_3_ [24,25]. The most significant mass loss is then observed between 650 and 900 °C and is mainly associated with the decarbonation of CaCO_3_ along with the final decomposition of the remaining carbonates [24]. There is an exothermic peak around 800–820 °C, which can be attributed to the formation of a mixed Na–Ca–Mg phosphate phase, together with magnesium and calcium borates. This is confirmed by XRD analysis performed on the corresponding treated mixtures following the thermal pretreatment (Figure 4). A strong endothermic event then occurs above 850–900 °C, corresponding to the melting of the mixture. No clear onset temperature can be determined, as boron- and phosphorus-based oxides start melting at lower temperatures. The heatflow curves also show that the melting event is still ongoing at 1100 °C. A minor mass loss between 900 °C and 1100 °C is also observed and can be attributed to the onset of oxide evaporation.

#### 3.1.2. After Melt Quenching

**Figure 6:**
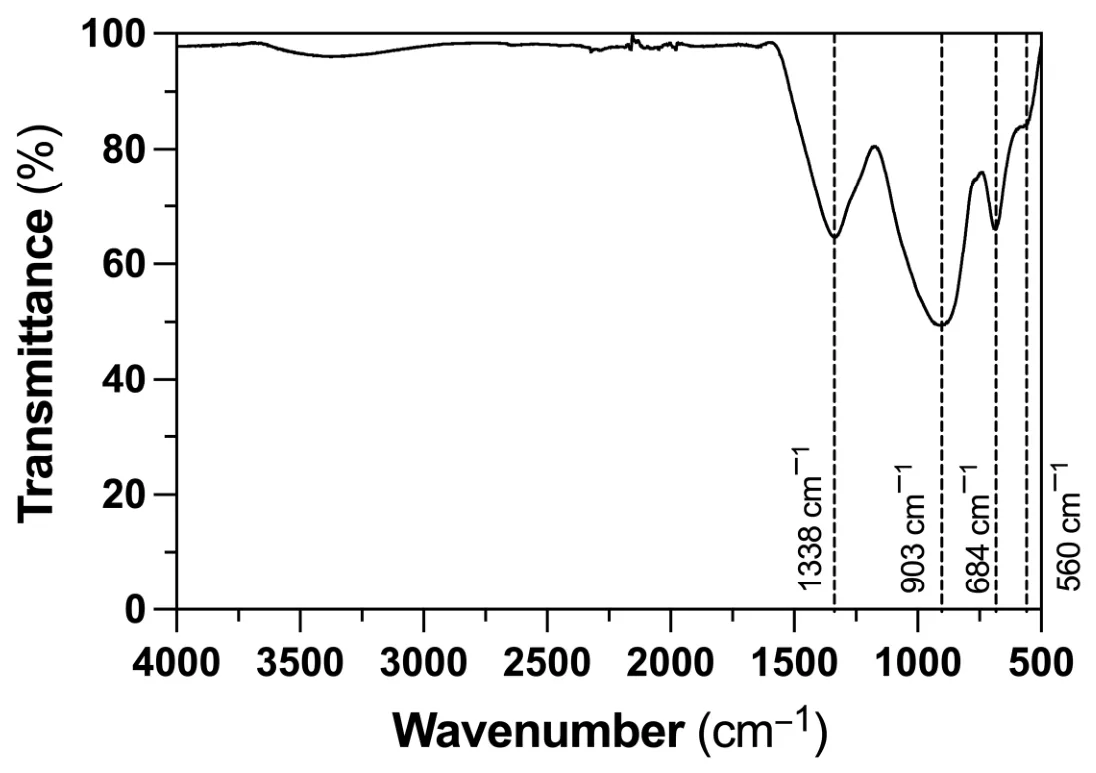
FTIR of the final borate-based bioactive glass obtained by melt quenching.

The FTIR spectrum shown in Figure 6 reveals three main absorption bands centered at 684 cm^−1^, 903 cm^−1^ and 1338 cm^−1^. A shoulder observed around 560 cm^−1^ can be attributed to P-O stretching of the PO_4_ units, while the band at 684 cm^−1^ corresponds to the B-O-B bending of BO_3_ units. The broadband centered at 903 cm^−1^ corresponds to B-O stretching vibrations of tetrahedral BO_4_ units contained in structures such as diborate and tetraborate [8,21]. Finally, the band at 1338 cm^−1^ is assigned to stretching vibrations of BO_3_ units containing non-bridging oxygens, resulting from the presence of network modifier Na_2_O [8,21]. Overall, this spectrum is characteristic of borate-based glasses and confirms the formation of a glass network with a high B_2_O_3_ content. However, the broad nature of the bands limits a more detailed structural analysis of the obtained glass.

**Figure 7:**
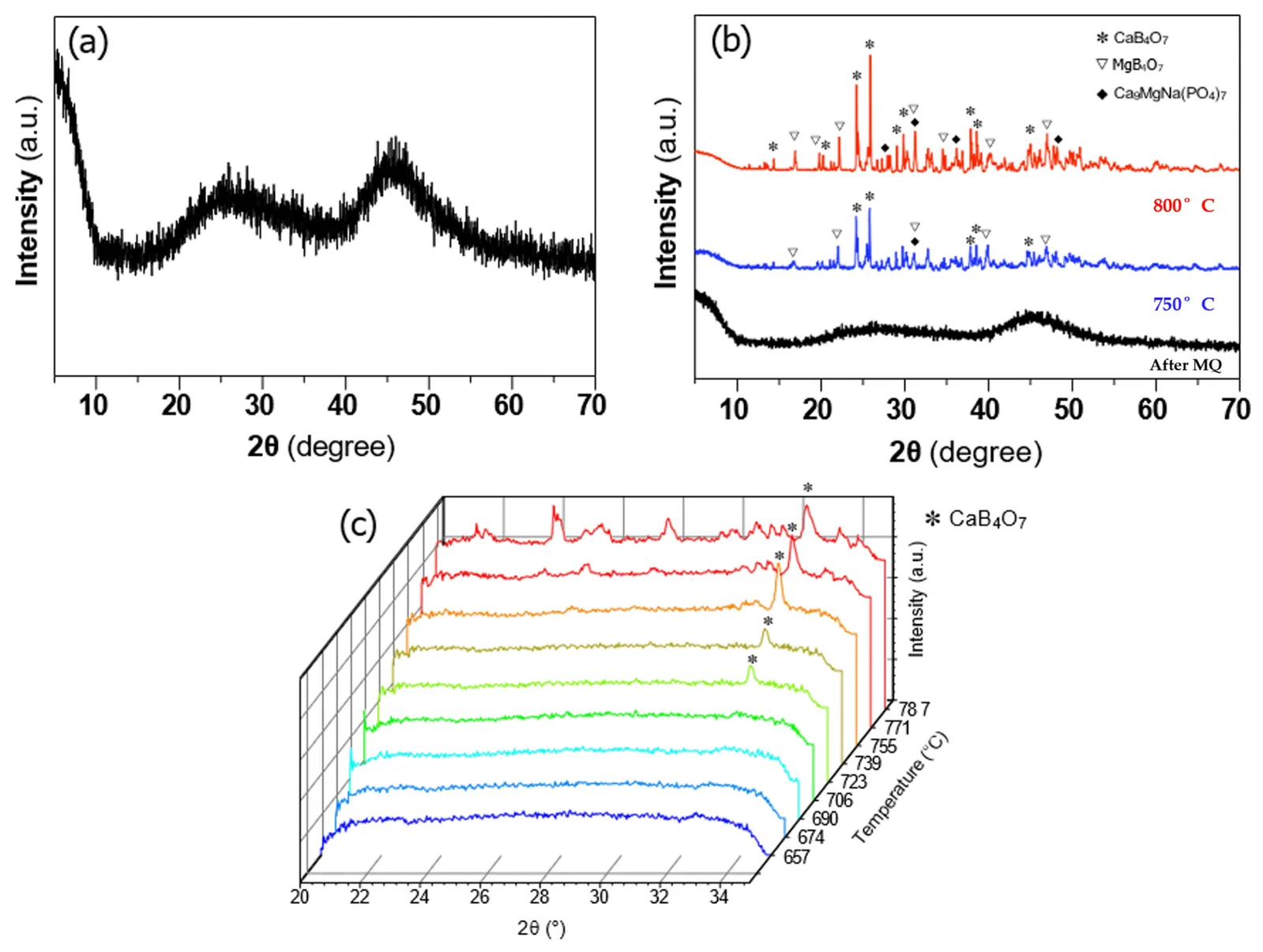
(**a**) XRD pattern of the final borate-based bioactive glass obtained by melt quenching, (**b**) XRD patterns after calcination at different temperatures (750 and 800 °C), and (**c**) in situ XRD patterns of the same glass in the 20–35° 2θ region. CaB_4_O_7_ (PDF 00-031-0253), MgB_4_O_7_ (PDF 00-031-0787), Ca_9_MgNa(PO_4_)_7_ (PDF 00-045-0136).

XRD patterns shown in Figure 7 provide a first structural assessment of the synthesized BBG. The amorphous nature of the glass is revealed in Figure 7a by the presence of two broad humps in the 20–35° and 40–55° 2θ regions, characteristic of borate glass networks. The first hump is commonly attributed to the combined contributions of network formers B_2_O_3_ and P_2_O_5_ while the second one is mainly attributed to B_2_O_3_ [32,33].

To investigate the incorporation of the oxides into the glass network and the onset of crystallization, further calcination treatments are performed at 750 °C and 800 °C for 30 min. The selected temperatures are based on the DSC results discussed above. As the temperature increases, diffraction peaks progressively emerge and become more defined. The XRD patterns in Figure 7b indicate the crystallization of calcium and magnesium borate phases, along with merrillite, a mixed Na-Ca-Mg phosphate phase. This evolution reflects the successful incorporation of the precursors into the glass network.

To get further insight into the crystallization process, in situ XRD measurements are carried out in the 20–35° 2θ region, where the most intense signatures are expected. As shown in Figure 7c, the onset of crystallization occurs between 706 °C and 723 °C, confirming the results from the TGA–DSC analysis. The appearance of a diffraction peak at approximately 32.5° 2θ is attributed to the formation of the CaB_4_O_7_ phase.

These results thus confirm that the “flash-melting” process leads to an amorphous BBG with a good integration of the different oxides into the borate network. In addition, they set an upper temperature limit for the sintering of the 3D-printed scaffolds to avoid crystallization.

To quantitatively justify how this specific synthesis route minimizes oxide evaporation and improves network homogeneity compared to conventional prolonged melt quenching, the thermal parameters were systematically optimized. Initially, increasing the target melting temperature to 1150 °C was required to lower the melt viscosity to a processable range. Despite achieving a lower melt viscosity at 1150 °C, the initial glass yield remained profoundly low (25–30%), accompanied by severe compositional drift driven by the unmitigated evaporation of volatile $B_{2}O_{3}$ and $P_{2}O_{5}$ fractions. To isolate the kinetics governing this event, the internal temperature profile of the furnace was measured directly using an independent thermocouple. The data revealed a substantial thermal lag: the furnace required approximately 2 h 45 min to ramp from 900 °C to 1150 °C rather than the expected 50 min. This extended residential period at elevated intermediate temperatures allowed extensive, uncontrolled oxide volatilization to occur. Based on these quantitative observations, the dwell time at peak temperature during standard ramping was progressively reduced to 0 min, which immediately restored the yield to 88% while preserving a low melt viscosity.

To completely eliminate the composition drift caused by the slow furnace ramp, a true “flash-melting” protocol was developed and screened across specific isotherm dwells at 1150 °C (10, 15, 20, and 30 min) by inserting the pretreated crucible directly into a preheated chamber. A dwell time of 20 min was selected as the definitive operational standard. This specific window uniquely balances processing efficiency and structural quality, achieving an approximately 100% material yield while providing sufficient time at high temperatures to ensure complete elemental blending and melt homogeneity prior to quenching.

The elementary composition of the synthetized amorphous borate-based bioactive glass is shown in Table 5 with the XRF analysis.

The experimental glass network is predominantly composed of boron trioxide (B_2_O_3_) measuring at 67.3 wt%, which closely matches the intended target of 68.1 wt%. The network modifiers (Na_2_O), CaO and MgO and the network former P_2_O_5_ all closely mirror the intended target values, showing only marginal deviations. The slight decrease in MgO from 4.9 to 4.3 wt% and P_2_O_5_ from 4.3 to 3.9 wt% can be attributed to minor volatilization or crucible interactions during the high-temperature melting process. As a direct result of this 20 min flash-melting optimization, the process restricted the volatilization loss of the dominant network former ($B_{2}O_{3}$) to a marginal relative deviation of just 1.17%, while the highly volatile $P_{2}O_{5}$ phase exhibited a relative deviation of only 9.30% (3.9 wt% experimental vs. 4.3 wt% target). This represents a significant quantitative advancement over conventional multi-hour inductive melt-refining setups reported in the literature [8,15,25,34], where unmitigated volatilization typically shifts the intended nominal targets by more than 5–10 wt%. Notably, while silica was completely absent from the initial batch formulation, the XRF analysis detected 2.6 wt% of ${\mathrm{SiO}}_{2}$ in the final glass. The minor incorporation of silica is an artifact of borate melt aggressiveness on silicate-based crucibles [34]. While the literature indicates that trace silicon substitutions in pure borate glass can structurally stabilize dissolution kinetics [18] and release silicon ions linked to osteoblast proliferation [12], further comparative dissolution and cytocompatibility studies are required to verify whether this minor 2.6 wt% fraction alters the biological or structural performance of these specific scaffolds compared to a completely silica-free matrix.

**Figure 8:**
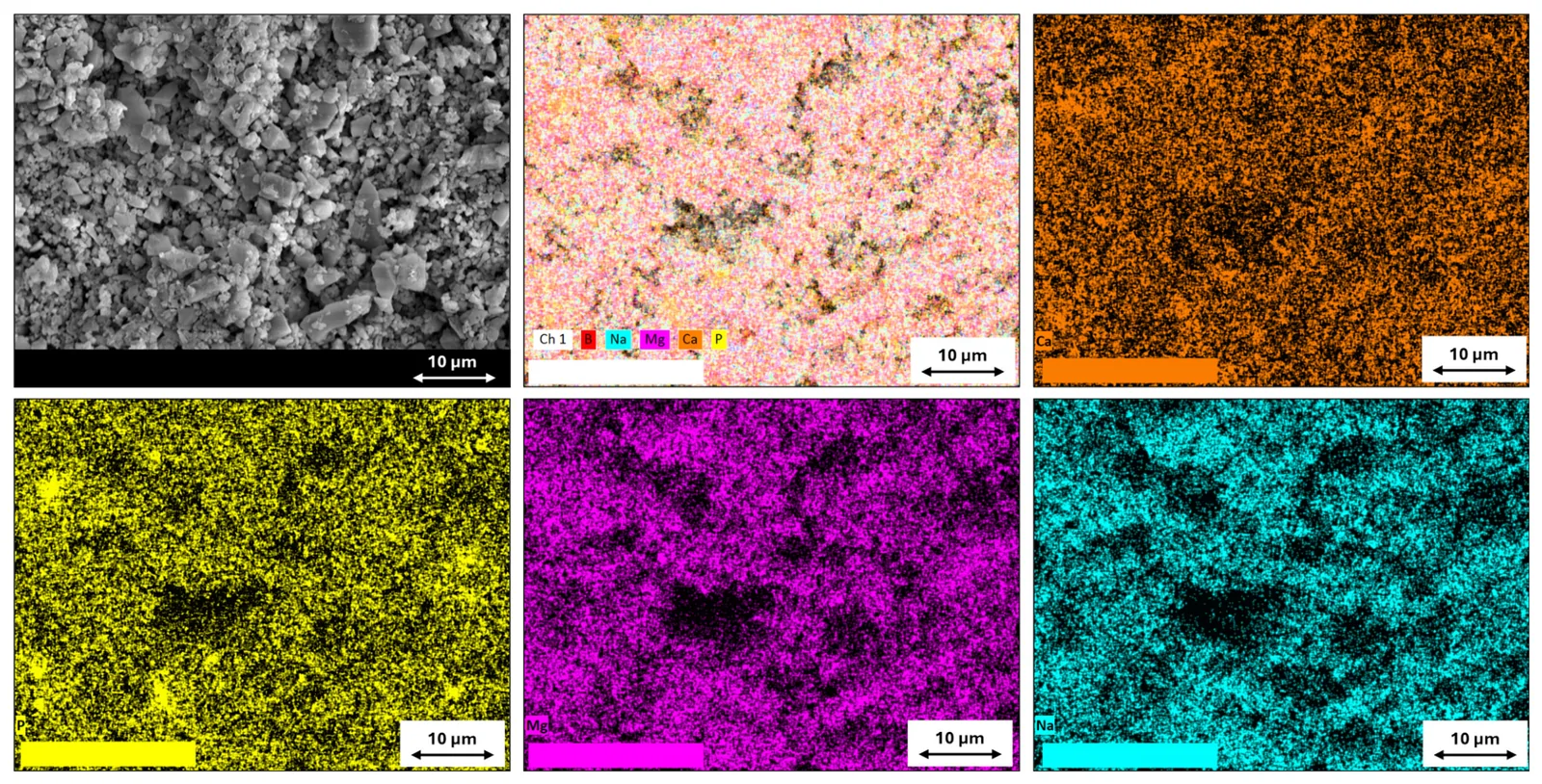
SEM micrograph and corresponding EDS elemental mapping of the final milled borate-based bioactive glass powder: (**top left**) secondary electron SEM micrograph showing particle morphology; (**top middle**) composite layered elemental map showing overall distribution; and individual elemental maps for (**top right**) calcium (Ca), (**bottom left**) phosphorus (P), (**bottom middle**) magnesium (Mg), and (**bottom right**) sodium (Na).

Microstructural and elemental spatial distribution of the final synthesized BBG powder is characterized by SEM-EDS elemental mapping (Figure 8).

The composite and individual elemental maps reveal that calcium (Ca, orange), phosphorus (P, yellow), magnesium (Mg, magenta), and sodium (Na, cyan) are homogeneously distributed throughout the entire particle field. The spatial intensity of each characteristic X-ray line directly maps onto the underlying solid particles, exhibiting complete overlap across both larger fragments and fine sub-micron debris. No elemental rich/depleted domains, phase separation, or unreacted precursor clusters are detected, visually confirming the high chemical homogeneity achieved through the combined spray-drying and flash-melting process.

**Figure 9:**
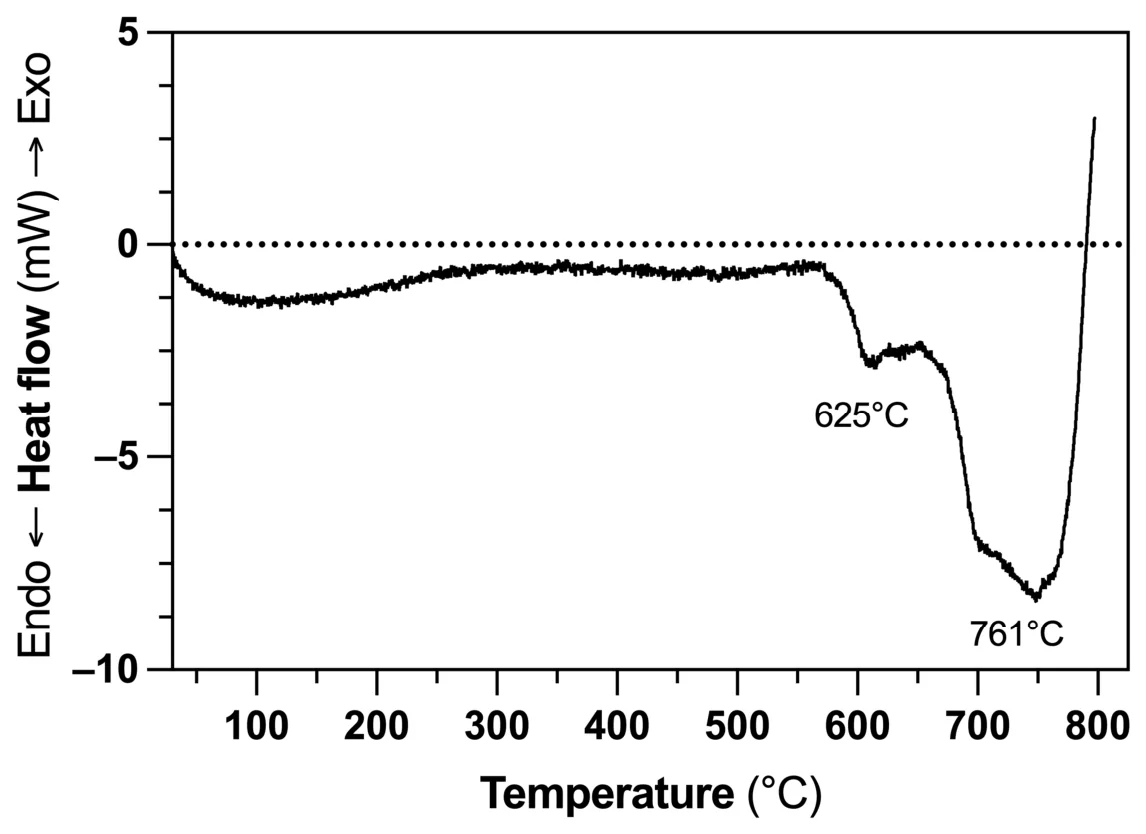
DSC curve of final composition.

Figure 9 presents the DSC curve for the obtained glass. A first endothermic event is observed at 625 °C, which can be attributed to the glass transition temperature (Tg). A second, more pronounced endothermic event occurs around 761 °C and is likely related to a structural rearrangement of the glass network. Such events are typical of borate glasses due to their unique complex structures. They correspond to a shift in the BO_4_ ←→ BO_3_ + NBO equilibrium (NBO = non-bridging oxygens), with a conversion of 4-fold coordinated boron to three-fold coordinated boron in the structures of short-range (BO_4_ and BO_3_ units) and intermediate-range (boroxol rings, tetraborate, diborate) orders [25]. These results are consistent with visual observations made during calcination trials on the glass powder. With increasing temperature, progressive densification followed by progressive vitrification and crystallization of the powder is observed around 675 °C up to temperatures above 700 °C.

**Figure 10:**
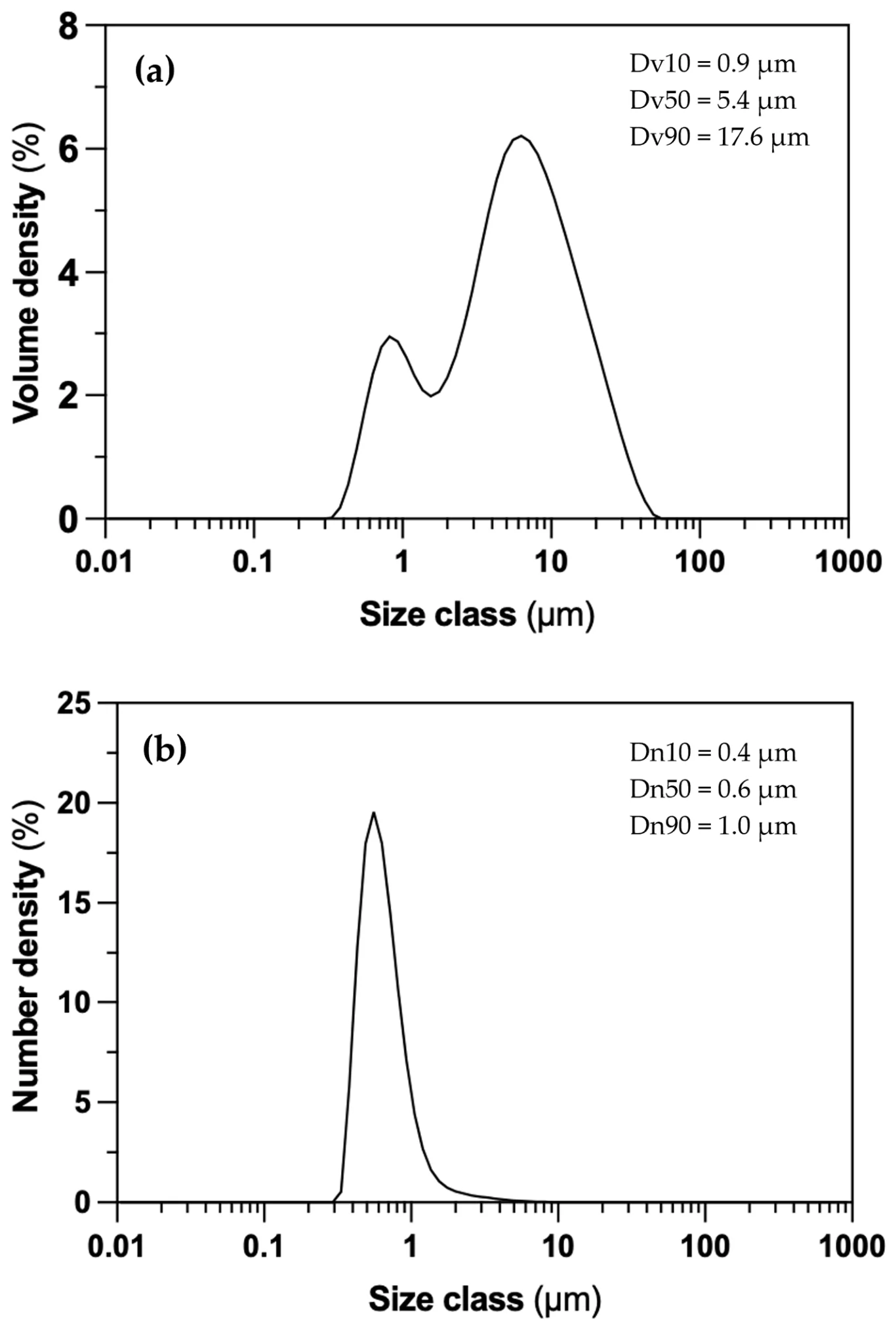
Granulometric analyses of the synthesized glass after planetary ball milling (2 × 15 min) and drying; (**a**) distribution in volume and (**b**) distribution in number.

Granulometric analyses of the synthesized glass after planetary ball milling (2 × 15 min) and drying are shown in Figure 10.

The first observation is that the planetary ball milling leads to a bimodal distribution in volume density, centered around 0.9 µm and 5 µm (Dv50 of 5.4 µm), together with an intense monomodal distribution centered at 0.6 µm in number density (Dn50 of 0.6 µm). This kind of distribution is optimal for the integration into a slurry matrix, as the smaller particles can fill the voids left by the bigger particles, allowing for a higher powder loading and thus a denser scaffold with a smoother surface after printing [29,35,36]. The final milled BBG powder is then compatible with DLP printing requirements.

**Figure 11:**
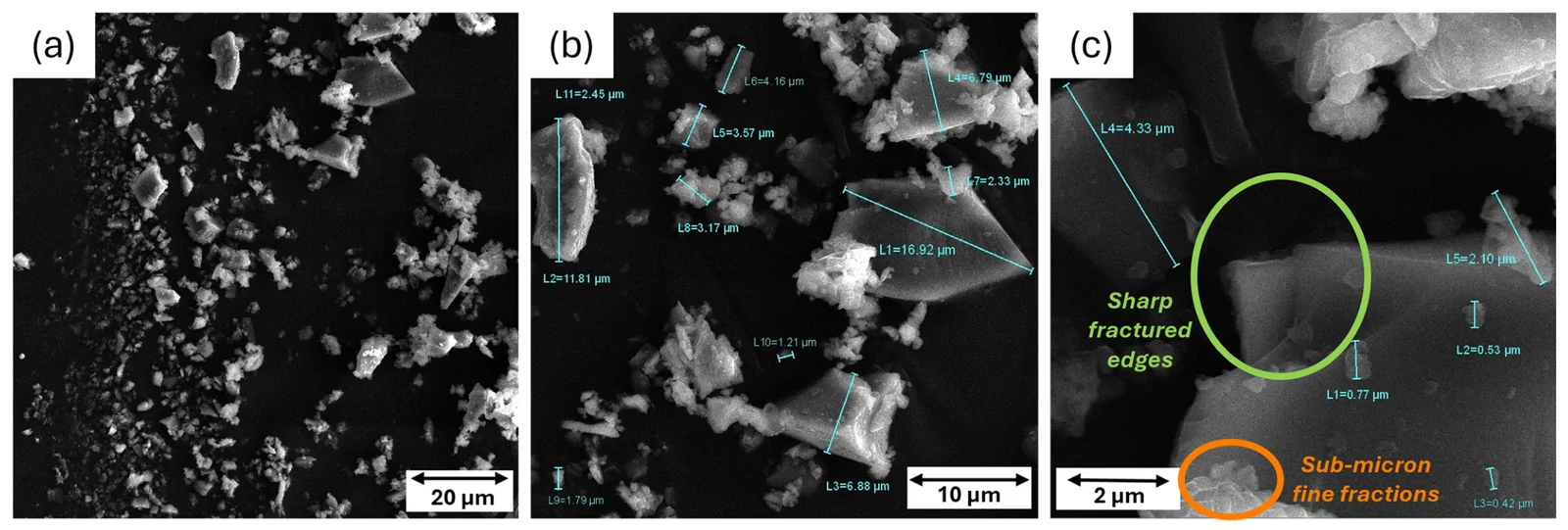
SEM micrographs of the milled synthesized BBG with measurements of the obtained particles.

Morphological analysis of the milled powder is depicted in Figure 11. The powder exhibits typical melt-quenched glasses morphology, with dense, non-porous particles with smooth surfaces and sharp edges. The size distribution matches the one obtained by laser granulometry, with a double population: bigger particles varying from a few microns to approximately 20 µm, with a high number of undefined grains below 1 µm, found on these bigger particles.

Regarding SBF testing, the study of the obtained glass bioactivity is evaluated by XRD and SEM. Before any characterization, it is observed that the pellets disaggregated during immersion, resulting in fine powder. This deagglomeration behavior is attributed to a dual mechanism: (i) the intrinsic high dissolution kinetics of the amorphous borate network, which undergoes rapid ion exchange in aqueous environments, and (ii) insufficient physical densification, as the pellets were evaluated in their cold-pressed, non-sintered state to isolate the raw powder’s bioactivity. Without high-temperature thermal necking to fuse the particle boundaries, the rapid fluid penetration and localized chemical dissolution of the borate matrix quickly overcome the weak mechanical compaction forces, causing the structural breakdown of the pellet.

**Figure 12:**
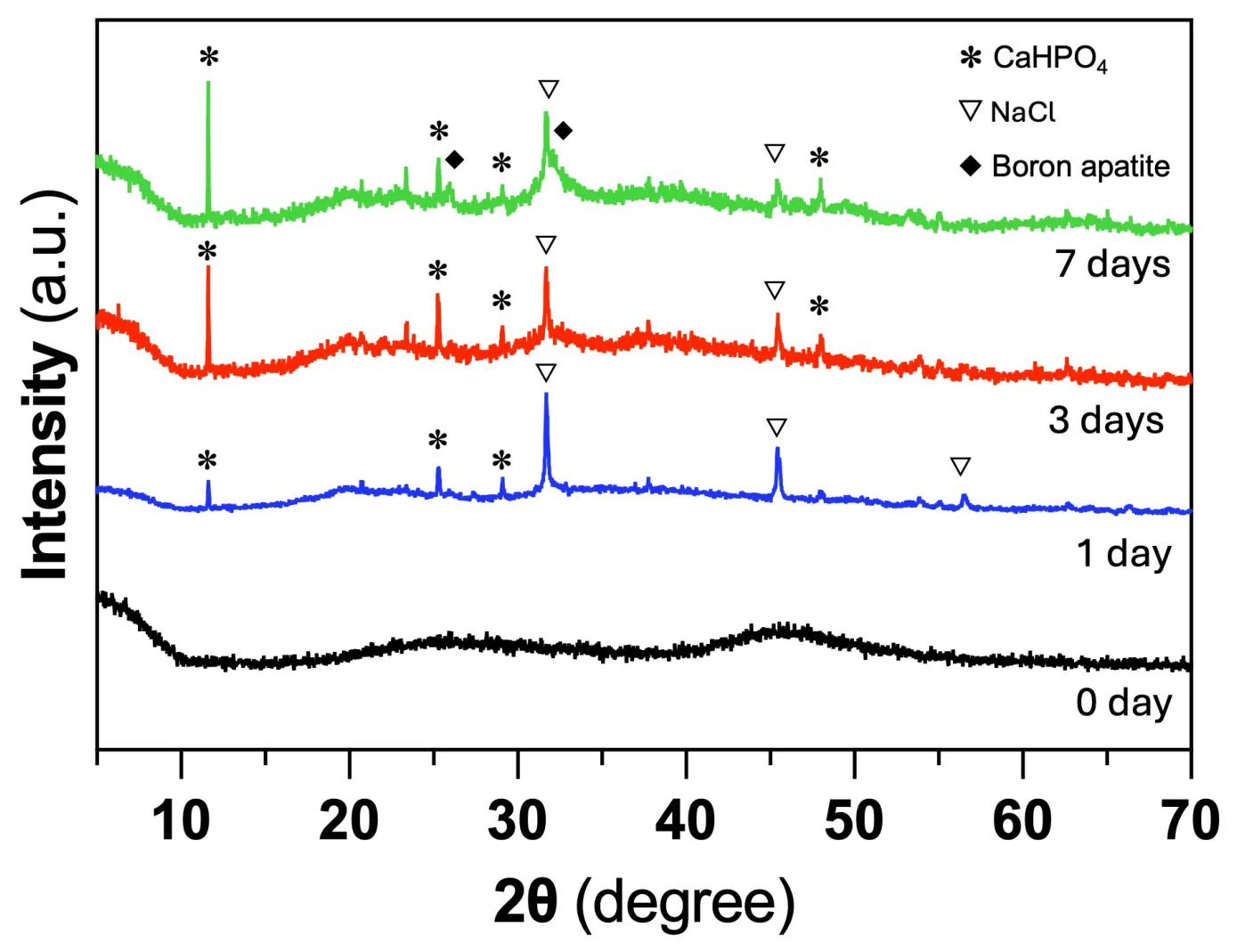
XRD patterns of synthesized glass after immersion in SBF for 0, 1, 3 and 7 days at 37 °C: CaPO_3_(OH) (PDF 00-011-0293), NaCl (PDF 01-080-3939), Boron apatite (PDF 01-080-0537).

XRD patterns of the glass before and after 1, 3 and 7 days of immersion in SBF at 37 °C are presented in Figure 12. Crystalline signatures already appear after 1 day of immersion, which is consistent with the high reactivity of BBGs [18]. The formation of a calcium phosphate phase (brushite) is detected from the first day of immersion and becomes progressively more intense with increasing immersion time. In addition, a boron apatite phase appears after 3 days of immersion and becomes more clearly defined after 7 days. The peak at 26° 2θ and the broad peak centered around 32° 2θ are characteristic of apatite-type phases [8,37]. NaCl diffraction signature is also observed and is likely due to NaCl precipitation in the SBF stock solution. These results confirm that the synthesized glass readily induces the formation of calcium phosphate phases in biological-like fluids, assessing its acellular bioactivity.

**Figure 13:**
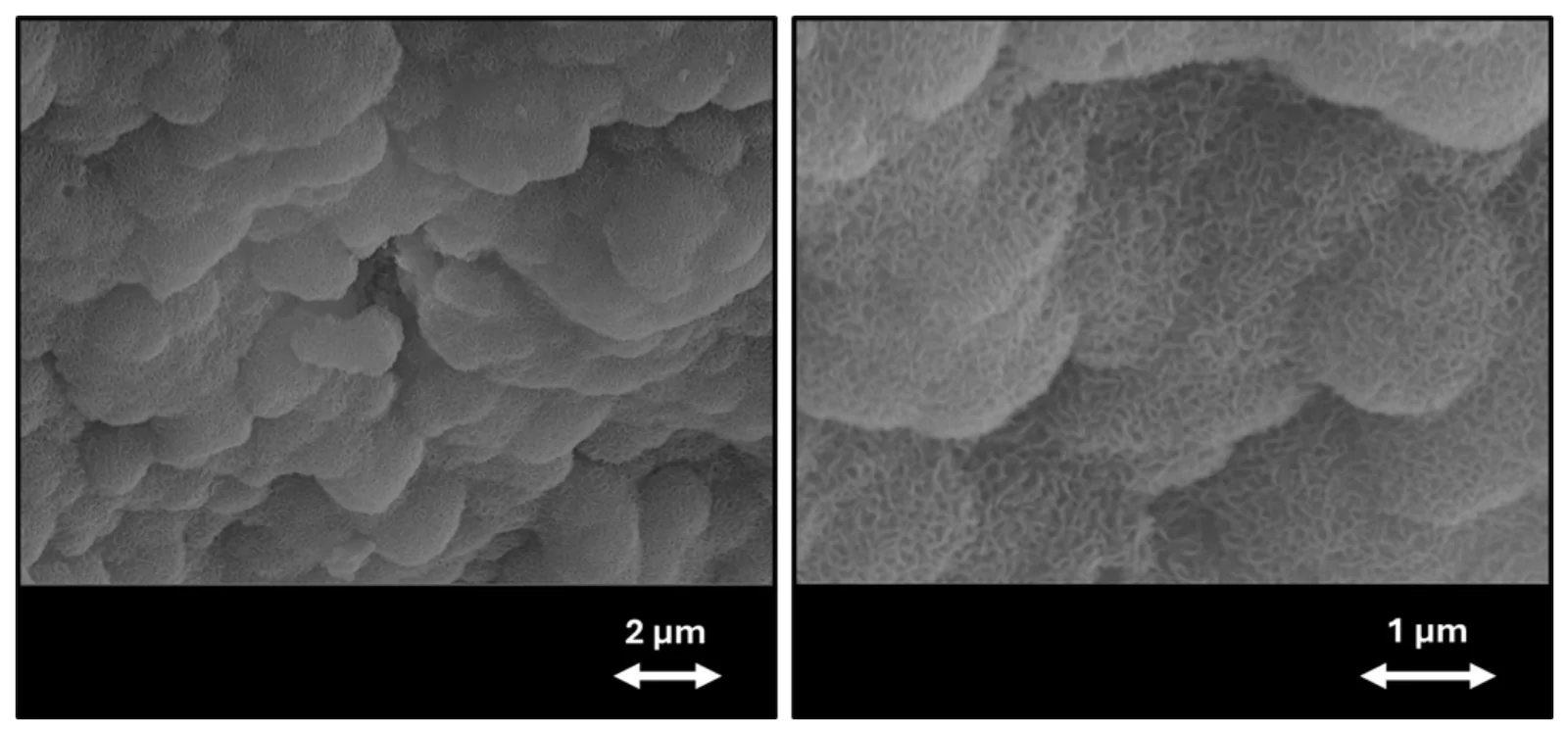
SEM micrographs of synthesized glass after immersion in SBF for 3 days.

The surface morphology of the powder after immersion in SBF for 3 days is shown in Figure 13. After immersion, the particles are fully covered by cauliflower-like structures of a few microns, composed of almost spherical sub-micrometric features. The cauliflower-like morphology is characteristic of the formation of an apatite phase, further confirming the results obtained by XRD measurements.

This Section 3.1. on the synthesis of a borate-based bioactive glass powder shows that a boron-based bioactive glass (BBG) powder is successfully synthesized via a spray-drying and “flash-melting” process, yielding a predominantly amorphous network with a minor silica incorporation (2.6 wt%) from crucible interaction that advantageously stabilizes dissolution kinetics. FTIR and XRD confirmed a well-integrated borate glass network characterized by a glass transition temperature (Tg of 625 °C) and an onset of crystallization between 706 and 723 °C, establishing an upper thermal limit for scaffold sintering.

The use of automated sub-micron spray drying allows us to achieve intimate pre-mixing of precursors, which drops the required melting time at 1150 °C to just 20 min. This rapid sequence restricts $B_{2}O_{3}$ loss to a minimal relative deviation of 1.17%. To provide a clear quantitative comparison with the literature, Table 6 introduces a comparative data matrix summarizing the processing times, temperatures, composition retention efficiencies (R), and residual species across distinct synthesis methods of borate-based bioactive glasses.

Following planetary ball milling, the BBG powder exhibits dense, non-porous morphologies with a bimodal volume distribution (Dv(50) = 5.4 µm and Dn50 = 0.6 µm). This particle size distribution is highly optimal for slurry formulation, promoting high powder loading and dense particle packing suitable for DLP 3D printing requirements.

Upon immersion in simulated body fluid (SBF), the powder demonstrates high reactivity, inducing the formation of a calcium phosphate phase (brushite) within 1 day, followed by a distinct boron apatite phase by day 3. SEM confirms this bioactivity through the rapid development of characteristic cauliflower-like apatite structures on the particle surfaces. Because the primary objective of this manuscript was to validate the technical manufacturing window (from raw powder engineering to high-resolution DLP gyroid printing), SBF was utilized as an immediate chemical proof-of-concept to verify that the high thermal exposure during flash melting did not deactivate the glass network. Comprehensive cell culture models (MTT viability and osteoblast seeding assays) are necessary as a biological follow-up study.

The next section presents the results of the produced BBG powder dispersion into a photo-curable resin and its 3D printing into gyroid scaffolds for bone implants.

### 3.2. Vat Photopolymerization of Borate-Based Bioactive Glass

**Figure 14:**
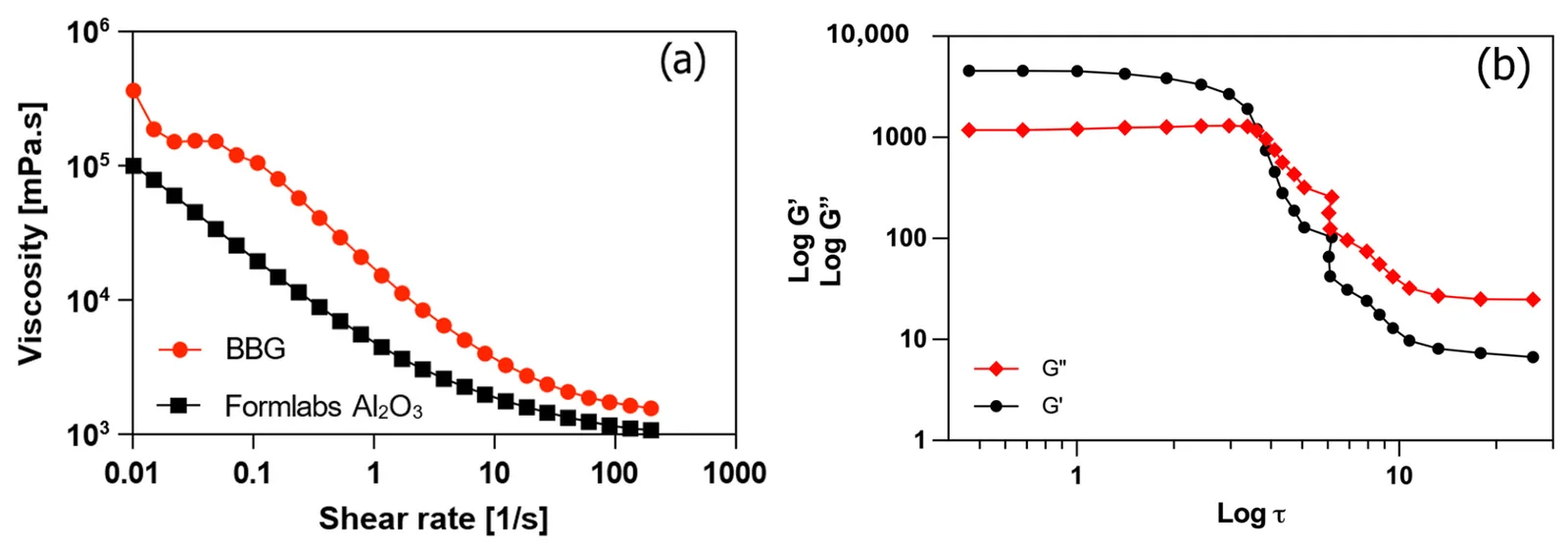
(**a**) Viscosity and (**b**) viscoelasticity curves of the prepared slurry.

The viscosity curve of the developed BBG slurry is presented in Figure 14a and compared with that of the commercial Alumina 4N Resin (Formlabs, Somerville, MA, USA). Although the BBG slurry exhibits a higher viscosity over the whole shear rate range, it shows the shear-thinning behavior required for DLP-based printing processes.

The higher viscosity at low shear rates, together with the presence of a small viscosity plateau, suggests the existence of a coherent internal structure at rest. This behavior suggests good suspension stability with limited particle sedimentation during printing. At a shear rate of $100\;s^{-1}$ (corresponding to the shear rate of the DLP recoating blade), both slurries exhibit similar viscosities, confirming that the rheological properties of the BBG slurry are compatible with the printing process. This shear-thinning drop ensures that the resin can flow smoothly across the vat floor without applying destructive hydrodynamic shear stresses on the delicate features of the freshly cured gyroid layer.

The viscoelastic properties of the slurry are shown in Figure 14b. At small shear stress, the storage modulus G′ is higher than the loss modulus G″, indicating an elastic behavior and a coherent internal structure. This high rest elastic modulus mathematically supports the high solid loading of dense glass microparticles (51.2 wt%) against gravity. Above a log τ value of 3.62 the trend reverses, indicating the collapse of the internal structure and that the slurry has transitioned from solid-like to fluid-like behavior. An LVER could be determined where G′ is quite constant at log τ values below 1. After this region, G′ decreases immediately upon increasing shear stress. This structural transition confirms a highly responsive yield response where the material remains structured and sedimentation-free at rest, but instantly breaks down into a low-resistance fluid the moment the printing mechanism initiates a recoating cycle.

**Figure 15:**
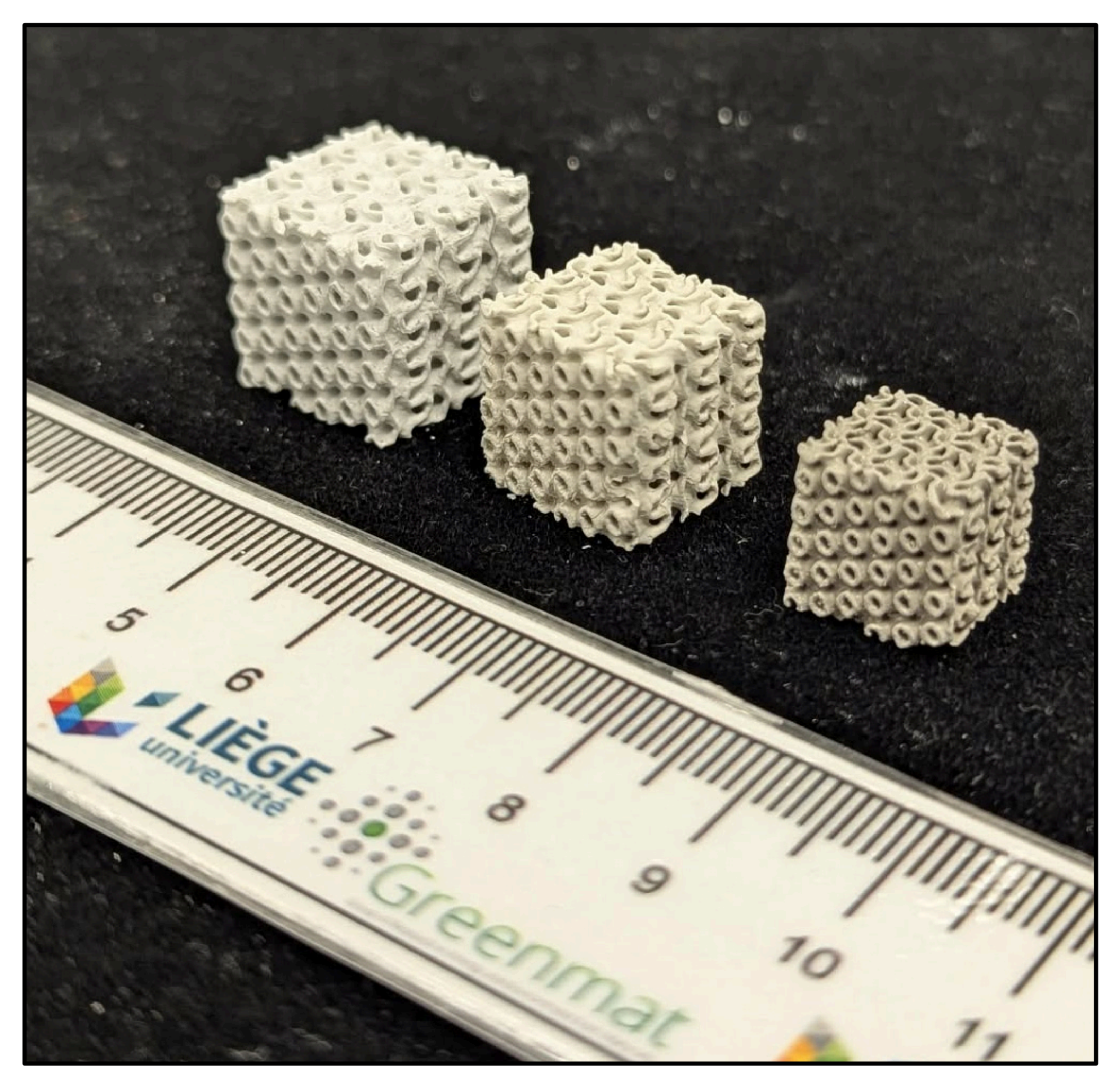
Photographs of as-printed part (**left**), debinded at 600 °C (**middle**) and sintered at 650 °C (**right**).

After rheological characterizations, gyroids cubes are printed using the BBG slurry and the STL model shown in Figure 2. A green part is visible in Figure 15 (left) along with a debinded part at 600 °C (middle) and a sintered one at 650 °C (right), showing a first qualitative assessment of the printing feasibility.

**Figure 16:**
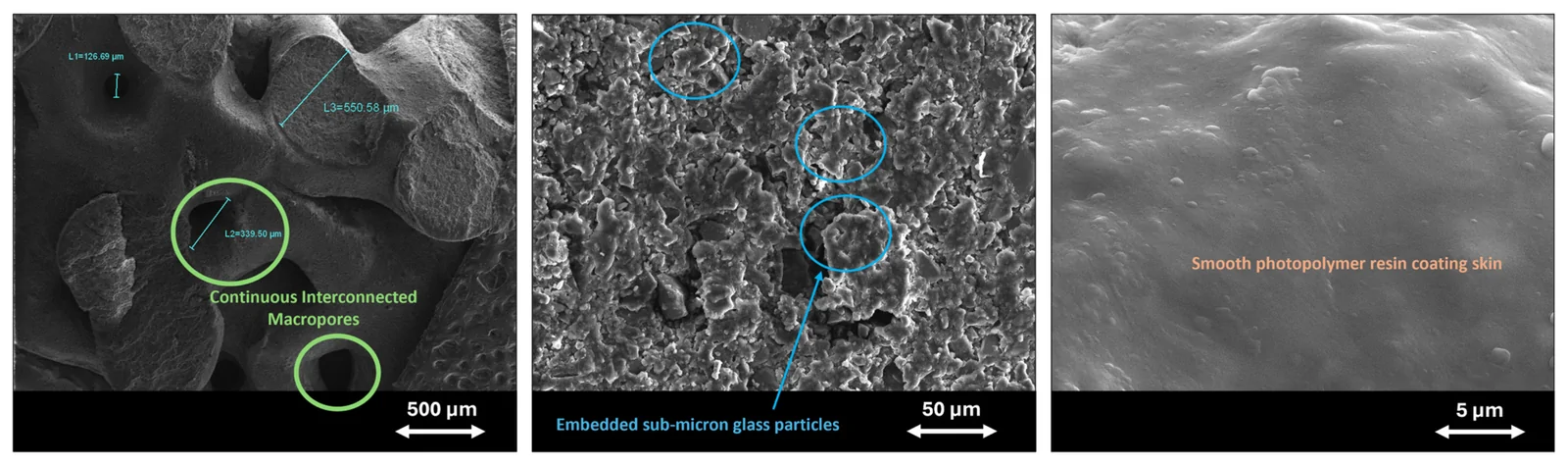
Micrographs of one BBG green part.

SEM pictures of the green part are shown in Figure 16.

The leftmost image provides a bird’s eye view of the printed scaffold’s architecture, showcasing its interconnected, open-pore network. The scaffold exhibits large, irregular, and highly interconnected macropores (around 330 µm). The struts are thick (around 550 µm) and continuous, showing that the DLP print maintained structural integrity prior to thermal processing.

The middle image zooms in on the surface of one of the structural struts, revealing the composite nature of the “green” state. The surface is highly granular and rough, composed of a dense distribution of borate bioactive glass microparticles embedded within a polymer matrix.

The rightmost image zooms in tightly on the outermost skin of the strut, highlighting the interface of the green body at a near-submicron level. The surface appears remarkably smooth, undulating, and continuous. This indicates that a thin layer of the cured photopolymer resin is coating the underlying bioactive glass particles. Small, rounded nodules and subtle bumps (ranging from tens of nanometers to about 1 µm) are visible just beneath or embedded within this polymer layer. These are the topmost edges of the finest borate glass particles embedded in the resin. The resin matrix appears homogeneous and free of visible microcracking or delamination, confirming a successful and uniform UV-curing phase during the DLP printing process.

**Figure 17:**
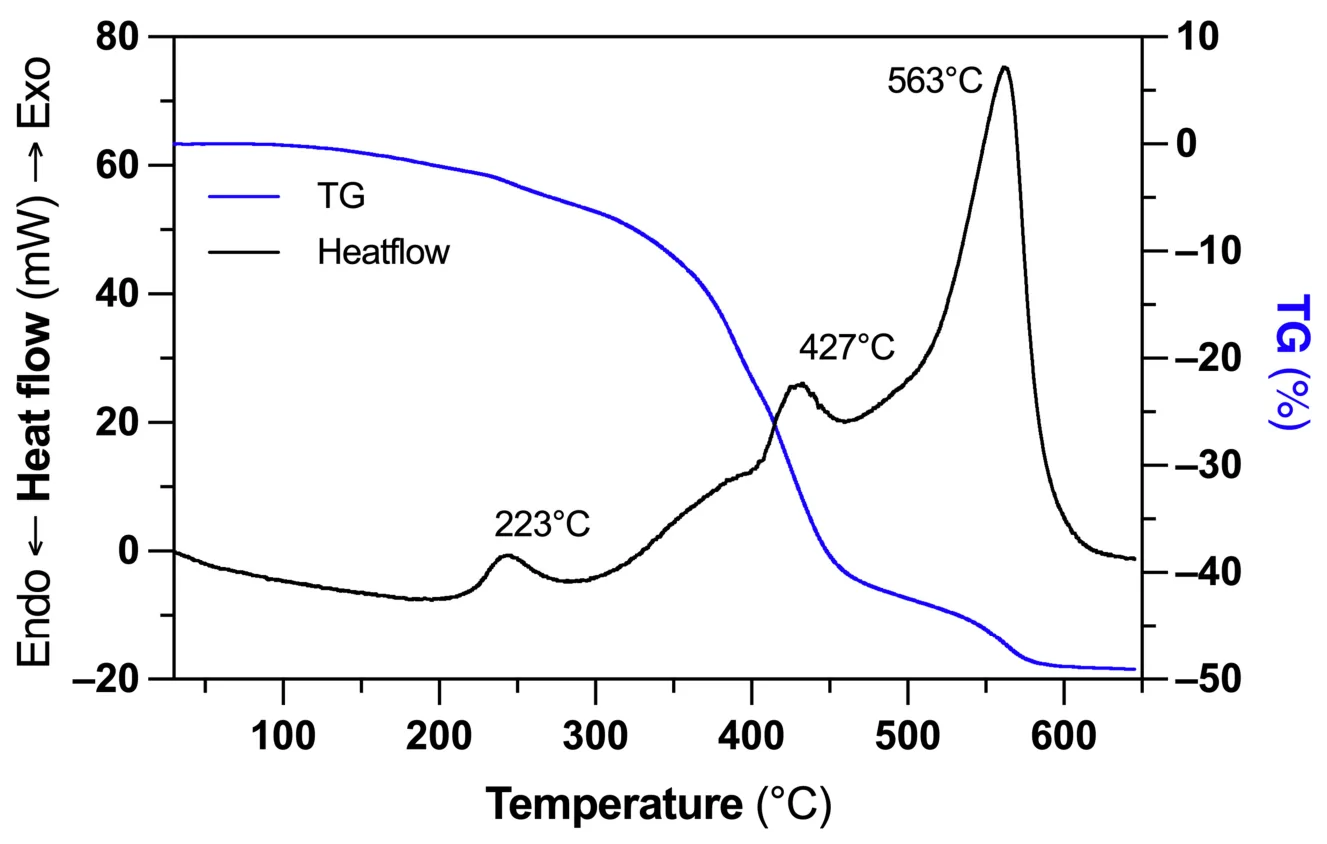
TGA-DSC curves of the printed green part.

The TGA-DSC analysis on the green part is presented in Figure 17.

First, the total mass loss is 49.1%, which is consistent with the slurry solid loading of 51.0%. A first minor mass loss occurs between 35 and 255 °C, coinciding with a weak exothermic event at 223 °C. This step is attributed to the decomposition of low-molecular-weight species, such as the plasticizer (PEG300) and the dispersing agent. The two additional mass losses observed between 255 and 600 °C, associated with the exothermic events centered at 427 °C and 563 °C, correspond to the degradation of the water-wash and high-speed polymerized acrylate resins, respectively. The high-speed resin degrades at a higher temperature because it contains pre-existing oligomer chains prior to curing, leading to the formation of longer and more thermally stable polymer networks after photopolymerization. Above 600 °C, the mass stabilizes, confirming the total elimination of all the organics and justifying the final temperature for the debinding cycle.

**Figure 18:**
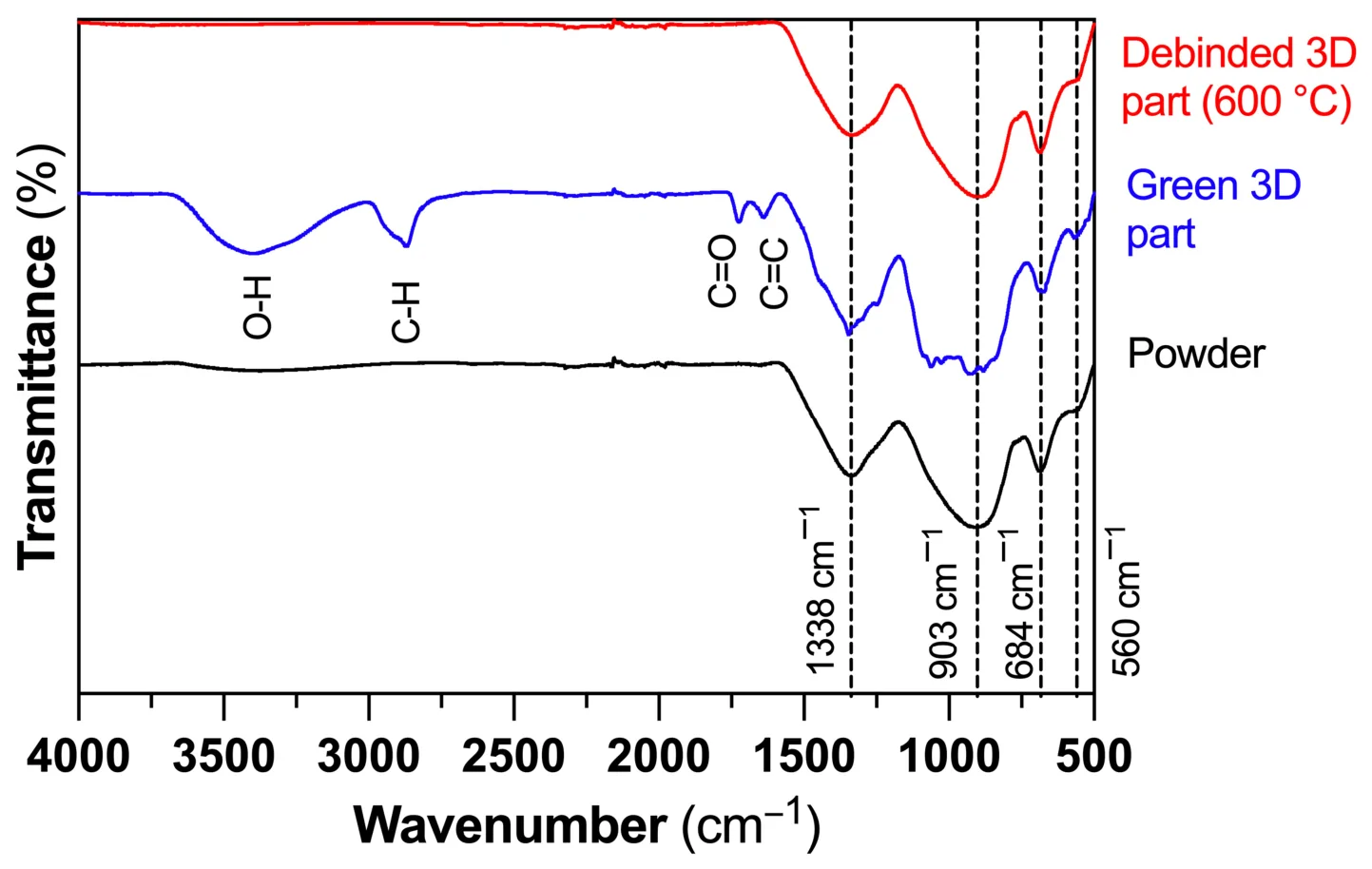
FTIR spectra of the final BBG powder, the green and debinded 3D parts.

Based on these TGA-DSC conclusions, green parts undergo a debinding treatment as detailed in Figure 3 (Section 2.3.3) up to 600 °C. FTIR analyses, shown in Figure 18, confirm the elimination of organic compounds during the debinding cycle. The characteristic bands of borate-based glasses, previously described in the characterization of the synthesized glass, are still visible in both the green and debinded bodies, indicating that the borate network is not altered by the printing or debinding steps. The spectrum of the green body shows typical signatures of organic compounds, including O-H stretching vibrations at around 3450 cm^−1^, C-H stretching at around 2900 cm^−1^, C=O stretching at 1740 cm^−1^ and C=C stretching at 1670 cm^−1^. The debinding treatment leads to a spectrum almost identical to the initial powder, consistent with the TGA–DSC results and confirming the effective removal of organic species.

**Figure 19:**
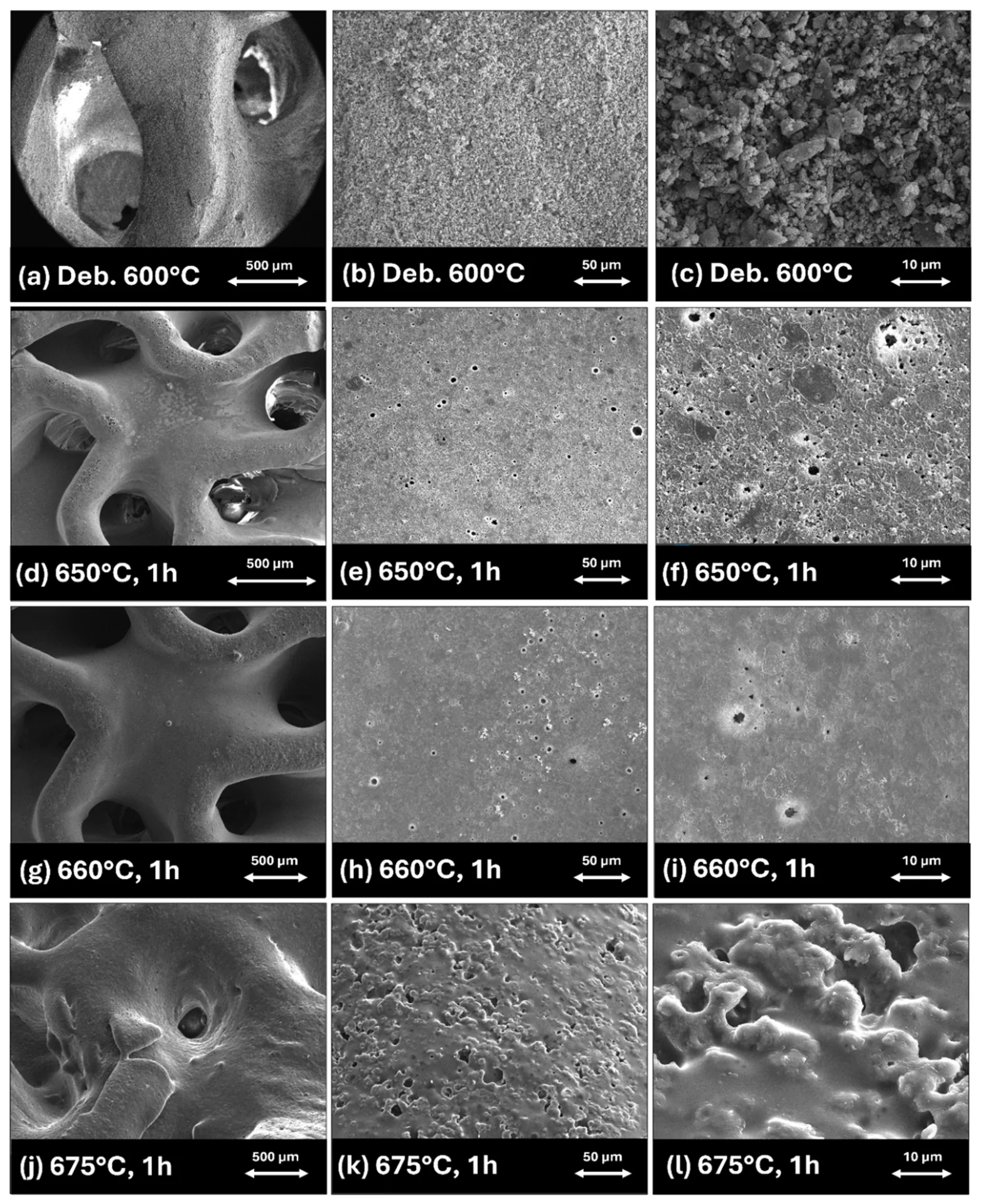
Micrographs of (**a**–**c**) a debinded BBG part at 600 °C; (**d**–**f**) sintered BBG part at 650 °C, 1 h; (**g**–**i**) sintered BBG part at 660 °C, 1 h; and (**j**–**l**) sintered BBG part at 675 °C, 1 h.

Following the debinding, different sintering cycles (650, 660 and 675 °C during 1 h, 300 °C/h) are tested and compared (Figure 3). SEM micrographs of the scaffolds at each stage (debinding at 600 °C and sintering at 650, 660 and 675 °C) are shown in Figure 19. This comprehensive SEM panel tracks the morphological evolution, densification, and sintering behavior of the 3D-printed borate bioactive glass scaffolds across different temperatures, progressing from the debinded state up to the glass melting point (around 675 °C).

The debinding stage (600 °C, Figure 19a–c) represents the scaffold after the photopolymer resin binder has been thermally removed, but before cohesive sintering has occurred. The macroporous, interconnected 3D-printed architecture remains intact, but the struts appear fragile and loosely held together. At higher magnifications, the microstructural texture is highly granular. Individual, irregular borate glass particles are distinctly visible with sharp edges. There is extensive interstitial microporosity between the loose particles, indicating zero densification at this stage.

At the initial sintering stage (650 °C for 1 h, Figure 19d–f) the borate glass reaches its glass transition temperature range, initiating viscous flow sintering. The struts smoothen significantly, taking on a rounded, shiny appearance. The interconnecting pore networks become clean and distinct. Individual glass particles have coalesced into a continuous matrix via viscous flow. However, densification is incomplete, as evidenced by a high density of micropores trapped on the strut surfaces.

Increasing the temperature by just 10 °C drastically improves the densification kinetics without destroying the structural architecture (660 °C for 1 h, Figure 19g–i). The scaffold maintains excellent structural fidelity. The struts are perfectly smooth, uniform, and structurally robust. The surface is significantly denser. The number of residual micropores has dropped drastically compared to the 650 °C treatment. The glass has flowed effectively to heal interstitial voids, representing the ideal sintering window where high densification is achieved while preserving the macroporous geometry.

At 675 °C (675 °C for 1 h, Figure 19j–l), the temperature reaches/surpasses the melting point of this specific borate glass composition (as shown in Figure 9 with the DSC analysis). Severe structural degradation is evident. The structural struts lose their mechanical stability and collapse or sag under gravity. The designed, interconnected macropores are clogging and closing up. The glass has turned highly fluid. It exhibits a glassy, reflowed morphology with large, irregular, smooth-edged craters and trapped gas bubbles. This indicates over-sintering, where low viscosity triggers structural collapse before the material can solidify into a stable form.

Based on these SEM observations, the ideal sintering temperature for this DLP-printed borate glass scaffold is 660 °C, balancing maximum particle densification (minimizing micropores) with the preservation of the macroporous 3D-printed architecture. Borate glasses exhibit a very narrow thermal processing window when a variation of only 25 °C shifts the material from under-sintered (650 °C) to completely melted/collapsed (675 °C).

**Figure 20:**
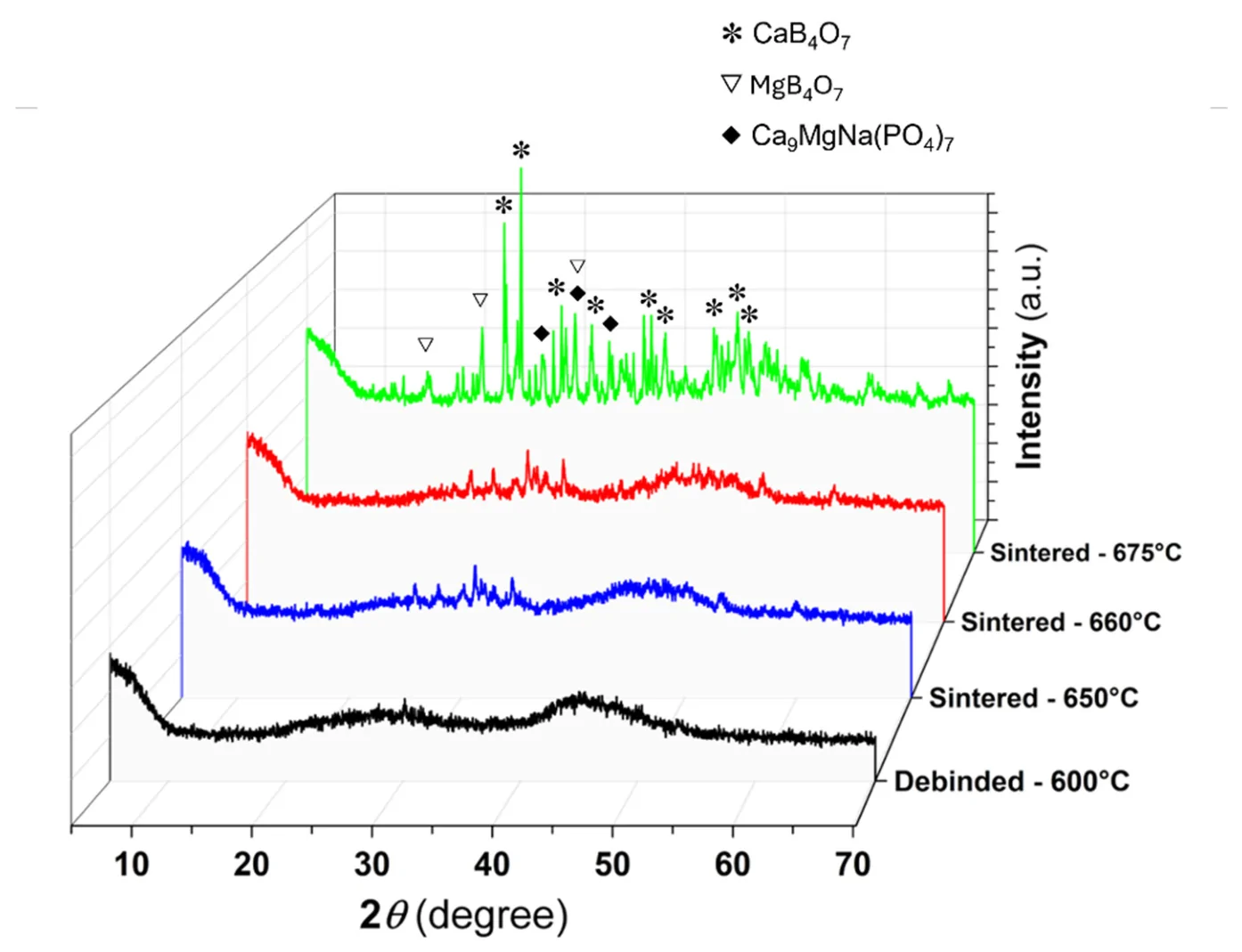
XRD patterns of the scaffolds after debinding at 600 °C and sintering at 650 °C, 660 °C and 675 °C for 1 h: CaB_4_O_7_ (PDF 00-031-0253), MgB_4_O_7_ (PDF 00-031-0787), Ca_9_MgNa(PO_4_)_7_ (PDF 00-045-0136).

XRD analyses are performed on the green, the debinded and the sintered bodies (600, 660, 650 and 675 °C) and are presented in Figure 20. The diffraction patterns reveal an onset of crystallization at lower temperatures than those observed after calcination of the powder and in situ XRD measurements. Indeed, the nucleation of the same phases (i.e., CaB_4_O_7_, MgB_4_O_7_ and Ca_9_MgNa(PO_4_)_7_) identified in crystallization tests already appears at 650 °C and is clearly confirmed at 675 °C.

This apparent shift compared to the in situ XRD measurement (Figure 7c) can be attributed to three main effects: kinetics, powder compaction, and the presence of defects. First, crystallization is a complex kinetic phenomenon. The slower heating rate used during sintering (300 °C/h compared to 600 °C/h for in situ XRD) combined with longer dwell times (1–2 h versus 30 min) promotes crystal nucleation and growth, even at lower temperatures [39]. Second, the compacted state of the powder after debinding, characterized by a more intimate contact between the particles, favors nucleation compared to loose powder. Finally, structural defects within the scaffolds (as observed by SEM) act as favorable nucleation sites [40]. These results show that crystallization is more condition-dependent rather than a fixed event for a specific material.

The dimensional variations and linear shrinkage behavior of the 3D-printed borate-based bioactive glass parts across different processing stages are summarized in Table 7.

Following the debinding stage at 600 °C, the parts exhibit isotropic contraction due to the thermal decomposition and removal of the organic binder matrix.

The subsequent sintering behavior shows a critical, highly sensitive dependence on the sintering temperature.

During the sintering at 650 °C, the parts achieve maximum volumetric contraction, with linear shrinkage values reaching 15.9%, 19.1% and 18. 3% relative to the debinded state in the different directions. This indicates significant particle rearrangement and initial-to-intermediate stage sintering driven by viscous flow.

At 660 °C, a noticeable reduction in shrinkage is observed and the geometric dimensions began expanding compared to the 650 °C condition, reaching only 12.3%, 12.7% and 17.5% of shrinkage relative to the debinded state (an expansion of 3.6%, 6.4% and 0.8%, respectively, relative to the 650 °C sintering).

The sintering treatment at 675 °C induces a severe expansion, yielding negative shrinkage values of −1.0% and −0.8% in two dimensions. This structural expansion indicates a classic de-densification phenomenon, typical of borate glasses due to high volatility and crystallization tendencies if the thermal treatment is not carefully controlled [8,39,41,42]. Indeed, the formation of crystalline phases during the sintering of borate glass (especially at slower heating rates) locks the microstructure and prevents efficient viscous flow, resulting in reduced densification or apparent expansion instead of shrinkage. Prolonged heat treatment or over-firing can lead to an increase in porosity [8,41], which is often caused by pore coalescence and the expansion of trapped gases [41]. Borate glasses, especially at high temperatures, can indeed experience weight loss through the volatilization of components like boron and sodium, which can cause internal expansion or gas entrapment [41,42].

To better understand the structural transformation during thermal processing, the relative density and porosity distributions are evaluated (Table 8).

As the temperature is raised from 650 °C to 675 °C, the relative density drops sharply from 63.9% to 30.7%, while the total porosity nearly doubles, increasing from 36.1% to 69.3%.

This degradation is primarily governed by the evolution of closed porosity, which expanded drastically from 13.0% at 650 °C to 48.4% at 675 °C. This behavior can be explained by two simultaneous mechanisms: viscous flow encapsulation and internal gas expansion (boron and sodium species) [8,39,41,42].

First, at higher temperatures, the rapidly decreasing viscosity of the borate glass causes surface pores to seal prematurely. This transitions the structural voids from open, interconnected channels into isolated, closed pores (viscous flow encapsulation) [41].

Second, residual carbonaceous species from the binder system or the localized volatilization characteristic of borate networks generates gaseous byproducts. Trapped inside the highly viscous melt, these gases expand to minimize internal pressure, causing the closed pores to bloat and forcing the macro-dimensions of the 3D-printed part to swell [42].

While open porosity remains relatively stable (fluctuating between 20.8% and 27.1%), the massive spike in closed pores establishes that an optimal sintering temperature could be between 650 and 660 °C. In this range, it would be possible to achieve maximum structural densification without severe over-firing and degradation of the mechanical integrity required for bioactive scaffolds.

As shown in Table 7, the scaffold sintered at 650 °C indeed exhibits a higher relative density (63.9%) compared to the 660 °C condition (49.4%). However, the SEM micrographs in Figure 19d–f clearly show that at 650 °C, viscous flow sintering is incomplete; individual glass particles are still poorly coalesced, and the structure is dominated by a vast network of sharp, interconnected interstitial micropores. When the temperature increases to 660 °C, the glass viscosity drops sufficiently to enable comprehensive particle necking and surface smoothing. Crucially, this advanced viscous flow begins to seal off external pathways, trapping residual binder outgassing and volatile glass species within the struts. This causes an expansion of closed pores (from 13.0% to 23.4%), which drives down the global bulk relative density via geometric swelling (Table 8).

Future works will be dedicated to the fine-tuning of debinding atmospheres and sintering windows (between 650 and 660 °C) to maximize the structural densification of the bioactive glass matrix without inducing premature, uncontrolled crystallization and porosity. The optimization of higher solid loading in the DLP resin could also help to produce scaffolds with improved density and higher mechanical strength. Finally, the progressive incorporation of additional materials, such as silicate-based bioactive glass (SBG), phosphate-based bioactive glass (PBG) and calcium phosphates could produce multi-material scaffolds. This strategy would allow control over bioactivity, mitigation of cytotoxic effects and optimization of the mechanical performance of the scaffolds.

A significant limitation of the current study is the absence of quantitative mechanical testing, such as compressive strength and elastic modulus determination. Due to the severe structural de-densification and spike in closed porosity (48.4%), the resulting scaffolds are mechanically fragile and unsuitable for immediate load-bearing bone applications. Consequently, these initial processing matrices are strictly limited to non-load-bearing bone graft configurations. Quantifying the compressive performance as a function of slurry solid loading and optimized vacuum-sintering schedules is an essential next step to bridge the gap between architectural printing feasibility and functional mechanical load-bearing engineering.

## 4. Conclusions

In conclusion, this study successfully demonstrates an integrated processing route mapping from raw chemical precursors to the additive manufacturing of biomimetic borate-based bioactive glass (BBG) scaffolds. The dual implementation of an aqueous spray-drying route and a precise multi-step thermal pretreatment efficiently prevents crucible foaming, reduces harmful material loss, and facilitates high yields during the subsequent 1150 °C flash-melting phase. Structural characterizations validate that the resulting low-sodium borate-based bioactive glass is completely amorphous and possesses highly reactive networks. This structural reactivity is confirmed by the rapid, biomimetic deposition of a crystalline apatite phase during in vitro SBF immersion assays, certifying its potential for bonding with hosting bone tissues.

Moreover, this study effectively transitions the optimized glass powder into advanced manufacturing by producing a stable, shear-thinning slurry with a 51.2 wt% solid loading. This formulation allowed the high-resolution printing of complex, interconnected gyroid architectures via Digital Light Processing. Although the printing feasibility of these biomimetic scaffolds has been robustly proven, post-processing challenges remain. The high residual closed porosity and early crystallization restrict the immediate mechanical viability of the scaffolds, representing a key weakness that must be resolved. Future efforts must focus on the fine-tuning of debinding atmospheres and sintering windows to maximize the structural densification of the BBG matrix without inducing premature, uncontrolled crystallization. Ultimately, this methodology offers a promising and highly reproducible proof-of-concept approach for creating custom-shaped, rapidly resorbable implants potentially tailored to patient-specific bone defects.

To bridge the gap between this initial processing validation and clinical realization, future research directions must prioritize three key vectors. First, mechanical optimization is required to overcome the strut de-densification noted during sintering; this will involve exploring higher initial powder solid loadings (>60 wt%) and vacuum-assisted thermal cycles to systematically eliminate closed microporosity. Second, a comprehensive biological evaluation must be established, shifting from acellular fluid models to in vitro cytocompatibility assays (e.g., cell viability, proliferation, and alkaline phosphatase expression using osteoblast lines) to directly monitor boron ion cytotoxicity. Finally, the long-term degradation behavior and structural degradation kinetics of these gyroid architectures must be evaluated under dynamic, biomimetic flow conditions to map how mass loss correlates with cell colonization and progressive mechanical softening over time.

## Figures and Tables

**Table 1:** Chemical precursors selected for borate-based bioactive glass synthesis.

Glass Oxide	Precursor	Purity and Supplier
B_2_O_3_	B(OH)_3_	≥99.8%, Carl Roth (Karlsruhe, Germany)
Na_2_O	Na_2_CO_3_	≥99.9%, anhydrous, Merck (Darmstadt, Germany)
CaO	CaCO_3_	≥99.9%, anhydrous, Sigma-Aldrich (St. Louis, MO, USA)
MgO	(MgCO_3_)_4_Mg(OH)_2_ · 5 H_2_O	Ph. Eurn, Carl Roth
P_2_O_5_	(NH_4_)_2_HPO_4_	≥98.0%, Sigma-Aldrich

**Table 2:** Spray-drying parameters.

Parameters	For This Study
Atomizing mode	Fountain (injection from the bottom)
Atomizing gas pressure	1 bar (air)
Feed rate	50 mL/min
Inlet temperature	255 °C
Outlet temperature	110 °C

**Table 3:** Composition of the DLP-compatible slurry.

Component	Reagent	Supplier	Ratio (wt%)
Powder	BBG	/	51.2
Resin 1	Water-Wash 2.0	Anycubic	28.0
Resin 2	High-Speed 2.0	Anycubic	4.5
Dispersing agent	Rhodafac^®^ RS-610 E	Syensqo	1.3
Plasticizer	PEG 300	Sigma-Aldrich	15.0

**Table 4:** DLP parameters to print BBG scaffolds.

Printing Parameters
Layer thickness: 0.05 mm
Operating temperature: 35 °C
Irradiance: 5.0 mW/cm^2^
Energy density: 30.0 mJ/cm^2^
Wiping speed: 100 mm/s
Rest time between layers: 0.50 s
Lifting speed: 60 mm/s

**Table 5:** Comparison of the targeted glass composition with the composition measured by XRF on the synthetized amorphous borate-based bioactive glass.

	Chemical Composition (wt%)					
	B_2_O_3_	Na_2_O	CaO	MgO	P_2_O_5_	SiO_2_
Targeted composition	68.1	3.8	18.9	4.9	4.3	/
BBG	67.3	3.7	18.2	4.3	3.9	2.6

**Table 6:** Data matrix summarizing the processing times, temperatures, composition retention efficiencies (R), and residual species across distinct synthesis methods of borate-based bioactive glasses.

Processing Parameter	Traditional Melt Quenching [8,21,38]	Standard Sol–Gel Pathway [7,31,33,38]	This Work (Spray Drying + Flash Melting)
Synthesis Temperature	1200–1400 °C	60–80 °C (aging) + 600 °C (calcination)	1150 °C
Total Processing Time	2–4 h	48–120 h (gelling/aging/drying)	<1 h (20 min melt)
$B_{2}O_{3}$ Composition Retention (R)	~90.0–93.0% (high volatization)	Variable (loss during washing/filtering)	98.83% (minimal volatilization)
Residual Carbonates/Nitrates	None (decomposed at high temp)	Risk of toxic residual nitrates	Completely eliminated at 800 °C

**Table 7:** The dimensions of the debinded and sintered bodies compared to the 3D file. Dimensions have been measured on 3 samples of each composition. The average standard deviation is around 0.2 mm for all samples.

3D Parts	Dimensions (mm)	Shrinkage Compared to Debinded Parts
3D file	15.60 × 15.60 × 15.34	
Green part	15.43 × 15.32 × 15.17	
Debinded (600 °C)	13.68 × 13.67 × 13.50	
Sintered 650 °C, 1 h	11.50 × 11.06 × 11.03	15.9% × 19.1% × 18.3%
Sintered 660 °C, 1 h	12.00 × 11.94 × 11.14	12.3% × 12.7% × 17.5%
Sintered 675 °C, 1 h	13.82 × 13.78 × 13.47	−1.0% × −0.8% × 0.2%

**Table 8:** Density and porosity values for the different sintering treatments. Average of 3 samples for each treatment with standard deviation (S.D.) of 1% for R.D. values and 0.1% for porosity values.

3D Parts	Relative Density (%)	Open Porosity (%)	Closed Porosity (%)	Total Porosity (%)
Sintered 650 °C, 1 h	63.9	23.1	13.0	36.1
Sintered 660 °C, 1 h	49.4	27.1	23.4	50.6
Sintered 675 °C, 1 h	30.7	20.8	48.4	69.3

## Data Availability

The original contributions presented in this study are included in the article. Further inquiries can be directed to the corresponding author(s).

## Abbreviations

The following abbreviations are used in this manuscript:

BG	Bioactive Glass
BBG	Borate-Based Bioactive Glass
SBG	Silicate-Based Bioactive Glass
PBG	Phosphate-Based Bioactive Glass
DLP	Digital Light Processing
SBF	Simulated Body Fluid
HAP	Hydroxyapatite
PLA	Polylactic Acid
PCL	Polycaprolactone
FTIR	Fourier-Transform Infrared Spectroscopy
XRD	X-ray Diffraction
XRF	X-ray Fluorescence
SEM	Scanning Electron Microscopy
DSC	Differential Scanning Calorimetry
TGA	Thermogravimetric Analysis
JCPDS-ICDD	Joint Committee on Powder Diffraction International Center for Diffraction Data
ATR	Attenuated Total Reflectance
LVER	Linear Viscoelastic Region
PP	Parallel Plate
RD	Relative Density
